# Proteomic Analysis Identifies Markers of Exposure to Cadmium Sulphide Quantum Dots (CdS QDs)

**DOI:** 10.3390/nano10061214

**Published:** 2020-06-22

**Authors:** Valentina Gallo, Vaibhav Srivastava, Vincent Bulone, Andrea Zappettini, Marco Villani, Nelson Marmiroli, Marta Marmiroli

**Affiliations:** 1 Department of Chemistry, Life Sciences and Environmental Sustainability, University of Parma, 43123 Parma, Italy; valentina.gallo@unipr.it (V.G.); nelson.marmiroli@unipr.it (N.M.); 2 Royal Institute of Technology (KTH), Department of Chemistry, Division of Glycoscience, School of Engineering Sciences in Chemistry, Biotechnology and Health, AlbaNova University Center, SE-106 91 Stockholm, Sweden; vasri@kth.se (V.S.); bulone@kth.se (V.B.); 3 ARC Centre of Excellence in Plant Cell Walls and School of Agriculture, Food and Wine, The University of Adelaide, Urbrae, SA 5064, Australia; 4 Department of Nanomaterials, Institute of Materials for Electronics and Magnetism (IMEM)Department of Nanomaterials, National Research Council (CNR), 43124 Parma, Italy; andrea.zappettini@imem.cnr.it (A.Z.); marco.villani@imem.cnr.it (M.V.); 5 The Italian National Interuniversity Consortium for Environmental Sciences (CINSA), 43124 Parma, Italy

**Keywords:** baker’s yeast, proteomics, iTRAQ, engineered nanomaterials, quantum dots, glycolysis, oxidative phosphorylation, endoplasmic reticulum

## Abstract

The use of cadmium sulphide quantum dot (CdS QD)-enabled products has become increasingly widespread. The prospect of their release in the environment is raising concerns. Here we have used the yeast model *Saccharomyces cerevisiae* to determine the potential impact of CdS QD nanoparticles on living organisms. Proteomic analyses and cell viability assays performed after 9 h exposure revealed expression of proteins involved in oxidative stress and reduced lethality, respectively, whereas oxidative stress declined, and lethality increased after 24 h incubation in the presence of CdS QDs. Quantitative proteomics using the iTRAQ approach (isobaric tags for relative and absolute quantitation) revealed that key proteins involved in essential biological pathways were differentially regulated over the time course of the experiment. At 9 h, most of the glycolytic functions increased, and the abundance of the number of heat shock proteins increased. This contrasts with the situation at 24 h where glycolytic functions, some heat shock proteins as well as oxidative phosphorylation and ATP synthesis were down-regulated. It can be concluded from our data that cell exposure to CdS QDs provokes a metabolic shift from respiration to fermentation, comparable to the situation reported in some cancer cell lines.

## 1. Introduction

Engineered nanomaterials (ENMs) show novel and interesting physico-chemical properties that have stimulated their use in many products currently available on the market [1]. In the past decade, ENMs have become ubiquitous and a part of our daily life in the form of components of cosmetics, food packaging, drug delivery systems, therapeutics, electronic systems, biosensors, and many other daily products [2]. The value of the global nanocomposite market is predicted to reach $5.3 billion by 2021, with a compound annual growth rate of 26.7% [3,4].

Among the numerous types of ENMs, quantum dots (QDs) are nanocrystals of semiconducting materials measuring around 2–10 nm, composed of metals belonging to groups II-V or III-V of the periodic table. They consist of a coated semiconductor inorganic core to improve optical and electronic properties [5,6]. Owing to their narrow emission waveband, bright fluorescence tuneable according to their dimensions, high photo-stability and broad UV excitation, QDs were initially adopted in precision optical devices [7], solar cells [8], new generation LEDs and lasers [9,10]. More applications of QDs include medical diagnostic tools and imaging detection systems for biomarkers of cancer cells [11,12,13], immunoassays, and cancer therapy [14,15], as well as transport vehicles for DNA, proteins and drugs to degenerative cells [16,17,18,19].

There are several reports of QD’s impact on human cell lines, simple eukaryotes and plants, which correlate toxicity to the surface properties, size and functionalisation of the nanomaterials [20,21,22,23,24]. Paesano et al. (2016) reported that CdS QDs trigger apoptosis, increase ROS concentrations and modify the transcription of key genes in HepG2 liver cells [21]. Similar results have been reported upon in vivo and in vitro exposure of mice liver cells to CdTe QDs [25], and when exposing HL-7702, HepG2 and HEK-293 cell lines to CdTe/CdS core/shell QDs, respectively [26].

There is a need for a paradigm shift in nanotoxicology, as advocated by the US National Academies of Sciences (2007). Also, EU legislation promotes Intelligent or Integrated Testing Strategies (ITS) for chemicals and specifically for ENMs (REACH Directive 1907/2006). In general, toxicology regulations for the 21st century promote the use of more efficient and more ethical tests, and encourage identification of toxicity mechanisms to build evidence-based testing strategies, and promote the use of in vitro, high-throughput screening (HTS) using cell lines and model organisms such as *Saccharomyces cerevisiae*, which presents 20% homology with the human genome [27]. To explore the mechanism of ENMs toxicity, new in vitro and *in silico* approaches together with the application of HTS have been advocated [28]. In particular, “omics”-based platforms applied to model organisms have provided key information on the interaction between ENMs and living materials [20,29,30].

In this work, a comparative proteomic analysis was employed to reveal the mechanisms of toxicity of CdS QDs in *S. cerevisiae*. We first investigated the most significant responses of the yeast to sub-lethal concentrations of CdS QDs at 9 h by using 2D-gel electrophoresis (2D-PAGE) and mass spectrometry. This method was utilised in the first instance to allow direct comparison with the yeast data available in the literature, which are essentially based on the use of 2D-PAGE [31]. In a second, more rigorous approach, we performed a gel-free quantitative proteomic analysis based on the iTRAQ technology to circumvent the typical drawbacks inherent to the use of 2D-PAGE, such as the limited solubilisation and separation capacity of certain classes of proteins. Compared to 2D-PAGE, the use of the gel-free approach provides more robust quantitative information and allows the identification of a much higher number of proteins [32]. Our iTRAQ data allowed the identification of the proteomic alterations that take place when the yeast cells are exposed to CdS QDs. The most salient result is the observation of a metabolic shift from respiration to fermentation. Altogether the data presented pave the way for a better understanding of the detrimental effects that CdS QD ENMs have on living cells. 

## 2. Materials and Methods

### 2.1. Reagents and Standards 

All reagents and standards were purchased from Sigma-Aldrich (St. Louis, MO, USA) unless otherwise stated.

### 2.2. Synthesis and Characterisation of the CdS QDs

The synthesis and characterisation of water-soluble CdS QDs are reported in the Supplementary Materials (Figures S1 and S2). X-ray diffraction (XRD) Thermo ARL X’TRA (Thermo Scientific, SA—Switzerland) and high-resolution transmission electron microscopy (HR-TEM, FEI, Luxenbuog) showed that the average static diameter of the CdS QD nanoparticles was 5 nm, and the crystal structure was that of hexagonal wurtzite (ZnS) with approximately 78% Cd. The average particle size (dh) of the aggregates estimated by dynamic light scattering and zeta potential (ζ) measured in ddH_2_O were of 178.7 nm and +15.0 mV, respectively. These values changed in yeast extract-peptone-dextrose (YPD) medium to 545 nm and −11 mV, respectively. 

### 2.3. Yeast strains and Growth Conditions

*S. cerevisiae* strain BY4742 (MATα his3Δ1 leu2Δ0 lys2Δ0 ura3Δ0) was used in all experiments [33]. Cells were grown at 28 °C with shaking at 130 rpm in a YPD liquid medium (1% w/v yeast extract, 2% w/v peptone, 2% w/v dextrose) for 9 and 24 h, which corresponds to the log and stationary growth phases respectively. The culture medium was either used without supplementation, or supplemented with 0.25 mg L^−1^ nystatin, 100 mg L^−1^ CdS QDs, or 0.25 mg L^−1^ nystatin plus 100 mg L^−1^ CdS QDs. A prior complete analysis of the CdS QDs minimal inhibitory concentration was carried out, using concentrations ranging from 0 to 250 mg L^−1^ with and without nystatin [24]. Nystatin was added to facilitate the uptake of CdS QDs [23]. The purity of the cultures was monitored by optical microscopy.

### 2.4. Exposure of Yeast Cells to Different CdS QD Concentrations

After 24 h culture, optical densities were measured at 600 nm (OD_600_) using a Cary 50 UV-visible spectrophotometer (Varian, Agilent technologies, Torino, Italy), and the OD_600_ was adjusted to 1.0 with sterile water. The cells were then serially diluted tenfold and aliquots (4 µL) of each dilution were spotted onto 2% w/v SD-agar (6.7 g L^−1^ yeast nitrogen base w/v, glucose 2% w/v, histidine 20 mg L^−1^, leucine 120 mg L^−1^, lysine 60 mg L^−1^, uracil 20 mg L^−1^) or 2% w/v YPD-agar in the presence or absence of CdS QDs (25–200 mg L^−1^). Cell growth was monitored at 28 °C over two days of culture.

To determine the toxicity of the CdS QDs, growth curves were plotted using concentrations ranging from 0 to 200 mg L^−1^ in the presence and absence of nystatin. Yeast cells were grown starting from liquid cultures pre-grown for about 12 h in YPD until an OD_600_ of 14 was reached. The cells were subsequently diluted to an OD_600_ of 0.2 in 10 mL YPD medium supplemented with 25, 50, 100 or 200 mg L^−1^ CdS QDs and cultured at 28 °C under continuous shaking (200 rpm) for 48 h. 

### 2.5. Determination of ROS and Cell Viability by Flow Cytometry 

The peroxide-sensitive fluorescent probe 2′,7′-dichlorodihydrofluorescein diacetate (H_2_DCFDA; Molecular Probes) was used to assess the generation of intracellular reactive oxygen species (ROS). This compound is converted by intracellular esterases to 2′,7′-dichlorodihydrofluorescein, which is then oxidised by intracellular ROS to its highly fluorescent oxidised form (DCF). ROS generation was assessed by incubating yeast cells for 9 and 24 h in the presence and absence of 100 mg L^−1^ QDs, followed by the addition of 20 μM of H_2_DCFDA in the dark. After 30 min incubation, fluorescence was measured with a NovoCyte^®^ flow cytometer (ACEA Biosciences, Inc., San Diego, CA, USA). To distinguish living cells from dead cells, a second dye, propidium iodide (PI) (MP Biomedicals, LCC), was utilised. The signal from DCF was detected with a FITC (fluorescein isothiocyanate) band pass filter, and the events (50,000) and images recorded were processed using the NovoCyte^®^ Express software (ACEA Biosciences, Inc., San Diego, CA, USA). 

### 2.6. Protein Extraction and Quantification

Cells were sampled after 9 h culture for 2D-PAGE, and at 9 h and 24 h for the iTRAQ experiments. Cell pellets were collected by centrifugation, washed with cold distilled water and stored frozen at −80 °C. For protein extraction for 2D-PAGE, the cells were resuspended in 300 µL of cold denaturing isoelectrofocusing (IEF) buffer containing 7 M urea, 2 M thiourea, 2% CHAPS, 1% ampholytes (pH 3–10, GE Healthcare), and 75 mM DTT (added just before use) containing a protease inhibitor cocktail (Sigma, cat # P8215) [34]. For iTRAQ analysis, cells were resuspended in 250 µL extraction buffer containing 7 M urea, 2 M thiourea, 2% CHAPS, 20 mM Tris, and the protease inhibitor cocktail. Acid-washed glass beads were added to mechanically lyse the cells using a Thermo Savant FastPrep® Cell Disrupter (Qbiogene Inc. Carlsbad, CA, USA). The cells were homogenised by vortexing four times for 45 s (the samples were cooled on ice for 30 s between each vortexing step) in the presence of glass beads in a volume equivalent to that of the cell pellet. Glass beads, insoluble material and cell debris were eliminated by centrifugation for 30 min at 4 °C and 12,000 g. 

The concentration of proteins in the lysates was determined according to a modified Bradford assay after acidification of the sample buffer with 20 mM HCl [35]. Bovine serum albumin (BSA) was used as a standard. Further sample preparation depended on the subsequent step, i.e., 2D-PAGE analysis or iTRAQ labelling.

### 2.7. Separation of Proteins by 2D-PAGE and Identification by MALDI-TOF-TOF MS

Proteins were separated by IEF in the first dimension (pH 4–7) and 12% SDS-PAGE in the second dimension [34]. Quantification of each spot on 2D-PAGE, recovery of the spots and protein identification by MALDI-TOF/TOF MS were performed as detailed in Supplementary Materials, Sections S.3–S.5.

### 2.8. Trypsin Hydrolysis and iTRAQ Labelling

Acetone precipitation was performed to remove non-protein compounds from each sample. Six volumes of cold acetone were added to the solutions containing 100 µg protein and the mixtures were placed at −20 °C for 1 h to allow protein precipitation. The resulting precipitates were sedimented by low-speed centrifugation and used for iTRAQ analysis. One hundred microgram of proteins from each sample was solubilised in 0.05 M triethylammonium bicarbonate containing 1% sodium deoxycholate. Disulphide bonds were reduced for 1 h at 60 °C in the presence of 5 mM tris-(2-carboxyethyl)-phosphine, and the resulting free thiol groups were alkylated at room temperature for 15 min using methylmethanethiosulphonate (10 mM). The proteins were hydrolysed for 16 h at 37 °C in the presence of 5% trypsin in 50 mM triethylammonium bicarbonate. The solutions were acidified by the addition of trifluoroacetic acid (TFA) to a final concentration of 0.5% and centrifuged to remove sodium deoxycholate. The supernatants were transferred to new tubes and dried under vacuum (Qbiogene Inc. Carlsbad, CA, USA). The dried peptides from the yeast samples were dissolved in 100 μL of 250 mM triethylammonium bicarbonate in 75% (v/v) ethanol and transferred to different vials containing the different iTRAQ reagents (114–117; AB SCIEX, Foster City, CA, USA). After 1 h incubation at room temperature, the reactions were stopped by the addition of 100 μL Milli-Q water. The iTRAQ-labelled peptides were pooled, and the mixtures were dried under vacuum. iTRAQ labelling of the peptides from the different biological replicates was performed in the same conditions, except that the labels were inverted to reduce bias between samples.

### 2.9. Strong Cation Exchange (SCX) Fractionation of the iTRAQ-Labelled Peptides

The dried iTRAQ-labelled peptides were resuspended in 3 mL of sample-loading buffer (10 mM ammonium formate, 20% acetonitrile, pH 3.0) and loaded on a 1-mL NuviaTMS cartridge prepared according to the manufacturer’s instructions (BioRad) at 0.5 mL min^−1^ using a syringe pump. After sample loading, the cartridges were washed with 5 mL of sample loading buffer at 0.5 mL min^−1^ and peptide elution was performed at the same flow rate with consecutive 1.5-mL ammonium formate salt plugs at pH 3.0 (30, 50, 80, 100, 125, 150, 175, 200, 225, 250, 275, 300, 325, 350, and 400 mM in 20% acetonitrile). The eluent from each salt plug was dried using a SpeedVac centrifugal vacuum concentrator, and the peptides were purified on a PepClean C-18 column (Thermo Fischer Scientific, Rockford, USA) prior to MS analysis.

### 2.10. Nano-LC-MS-MS Analysis of the Strong Cation Exchange Fractions

Peptide analysis was performed by reverse-phase LC–electrospray ionisation–MS-MS using a nanoACQUITY Ultra Performance Liquid Chromatography system coupled to a Q-TOF mass spectrometer (Xevo Q-TOF, Waters, Milford, USA). The peptides purified by strong cation exchange chromatography were dissolved in 0.1% TFA and loaded on a C18 trap column (Symmetry 180 μm × 20 mm, 5 μm; Waters, Milford, USA) that was washed with 1% (v/v) acetonitrile and 0.1% (v/v) formic acid at 15 μL min^−1^ for 10 min. The peptides eluted from the trap column were separated on a C18 analytical column (75 μm × 100 mm, 1.7 μm; Waters, Milford, USA) at 350 nl min^−1^ using 0.1% formic acid as solvent A and 0.1% formic acid in acetonitrile as solvent B in a stepwise gradient: 0.1–10% B (0–10 min), 10–30% B (10–110 min), 30–40% B (110–120 min), 40–85% B (120–125 min), 85% B (125–130 min), and 85–0.1% B (130–135 min). The eluting peptides were sprayed in the mass spectrometer (capillary and cone voltages set to 4 kV and 35 V, respectively), and MS-MS spectra were acquired using automated data-directed switching between the MS and MS-MS modes using the instrument software (MassLynx V4.0 SP4). The five most abundant signals of a survey scan (350–1500 m/z range, 0.9-s scan time) were selected by charge state, and the collision energy was applied accordingly for sequential MS-MS fragmentation scanning (50–1800 m/z range, 0.9-s scan time).

### 2.11. Data Processing, Protein Identification, and Quantification

An extensive search was used to profile the MS data [36]. The MS raw data files were processed using Mascot Distiller (version 2.4.3.2, Matrix Science, London, UK). The resulting “mgf” files were converted into the “. mzXML” file format using msconvert [37]. The “.mzXML” files were searched by MyriMatch version 2.1.120 [38] and X!Tandem version 2011.12.01.1 [39] (LabKey, Insilicos, ISB, Seattle, WA) using the *S. cerevisiae* protein database and the following settings: trypsin specific digestion with two missed cleavages allowed, peptide tolerance of 100 ppm, fragment tolerance of 0.2 Da, iTRAQ 4-plex for peptide N-t and Lys as fixed modifications, and, in variable mode, iTRAQ 4-plex on Tyr, oxidised Met and methylthio on Cys. For quantitative analysis, all intensities of the iTRAQ reporter ions were extracted using the Trans-Proteomic Pipeline (TPP) tool Libra and the isotopic correction factors from the iTRAQ reagent manufacturer. Normalisation of iTRAQ channels was performed by summing all intensities of reporter ions in each iTRAQ channel (for peptides above the Libra probability cut-off) and equalising each channel contribution by dividing individual reporter ion intensities by the corresponding channel-specific correction factor. All “.pep.xml” files obtained from PeptideProphet were combined using iProphet [40]. A protein list was assembled using ProteinProphet [41], and the final protein ratios were calculated using Libra. In all searches, a concatenated target-decoy database-search strategy was used to check the false positive rate, which was found to be less than 1% in all cases. Peptide sequences were exported for each protein, with a protein and peptide probability cut-off of 0.95. Peptides matching two or more proteins (shared peptides) were excluded from the analysis. Proteins with no unique peptides, i.e., proteins identified by shared peptides only, were also excluded. A protein was considered as identified if it contained at least one unique peptide. Only proteins identified by two or more unique peptides were used for quantification. The method of Ross et al., (2004) was used for statistical analysis of the quantitative data [42]. Briefly, the 115/114, 116/114 and 117/114 ratios corresponding to each protein were calculated for each of the two biological replicates and log2 transformed to obtain a normal distribution. All the values in each comparison dataset were normalised to the median log values, and global means and standard deviations were calculated for each biological replicate. Proteins whose average ratios fell outside a standard deviation of ±1 from the global mean in two biological replicates were considered significantly enriched and chosen for further analysis.

### 2.12. Glyceraldehyde 3-Phosphate Dehydrogenase (GAPDH) Activity Assay

The activity of GAPDH was determined using a GAPDH Activity Assay Kit (Abcam, Cambridge, UK) following the manufacturer’s instructions. The assay is based on spectrophotometric measurement of NADH formation catalysed by GAPDH. Cultures were grown for 9 and 24 h in the presence and absence of 100 mg L^−1^ CdS QDs were diluted to the same OD_600_ value of 1. Forty-five microlitre was used for the assay and the reaction was run for 60 min at 37 °C. The absorbance of the reaction mixture was measured at 450 nm in kinetic mode using the iMark™ Microplate Absorbance Reader (Bio-Rad). GAPDH activity (U) was calculated as the amount of NADH produced (nmol) per unit of time (min) and was normalised to the protein content of the whole-cell lysate determined by the Bradford Protein Assay (Bio-Rad Laboratories Inc., Hercules, CA, USA). 

### 2.13. Data Mining and Analysis

All experiments were carried out in triplicate from independent yeast pre-cultures. After checking for normality and variance homogeneity in the dataset, a one-way analysis of variance (ANOVA) was applied, with a confidence interval (C.I.) of 95%. Statistical differences between means were deduced using the Bonferroni SHD post hoc test, applying a threshold of *p* < 0.005. The SPSS v23 software (http://www.ibm.com/analytics/us/en/technology/spss/) was used for all analyses. Venn diagrams were generated using Venny 2.0 (http://bioinfogp.cnb.csic.es/tools/venny/index.html). To visualise proteomic data, hierarchical clustering was performed using the heatmap.2 routine implemented in the R software (www.R-project.org/). The PANTHER (pantherdb.org/) software was used to search for gene enrichment, and the Gene Ontology (GO) database provided functional annotation for the differentially expressed proteins. For each GO category, Bonferroni correction and a two-tailed Fisher’s exact test were used. The proteins identified were then subjected to metabolic pathway enrichment analysis, which was conducted according to the instructions from the Kyoto Encyclopaedia of Genes and Genomes (KEGG) Pathway Database. 

## 3. Results and Discussion

### 3.1. Cell Growth in the Different Conditions Tested

**Figure 1:**
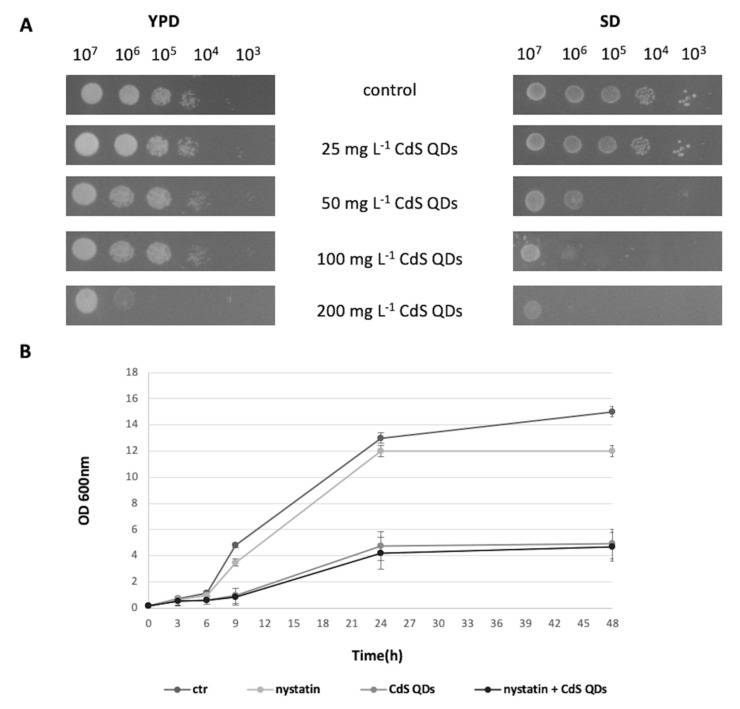
Spot assay and growth curve of *S. cerevisiae* cells. BY4742 grown on different media: YPD and SD. (**A**) growth at different cell dilutions as affected by the treatment conditions: control, 25, 50, 100, 200 mg L^−1^. Cell concentrations, used for the different tests, are indicated in the first row of the panel. (**B**) Growth curve of BY4742 with and without nystatin at 100 mgL^−1^ CdS QDs for 48 h.

*S. cerevisiae* strain BY4742 was grown on either YPD or SD medium in the presence of CdS QD concentrations of 25 to 200 mg L^−1^. The colony spot assay showed that yeast cells grew better on YPD than SD medium, therefore YPD was chosen for all subsequent experiments (Figure 1A). When nystatin was added at 0.25 mg L^−1^ [24], growth curves were comparable to the corresponding controls in YPD (Figure 1B). The concentration of 100 mg L^−1^ CdS QDs, with and without 0.25 mg L^−1^ nystatin, was chosen as the treatment for subsequent analyses [20,24]. The growth and treatment selected were identical to those used in previous transcriptomics analyses [20,23], allowing comparison between affected transcripts and proteins upon treatment with CdS QDs. Duration of the treatment was first set at 9 h, which corresponds to the exponential growth phase of the yeast cultures, and then at 24 h for the stationary phase. Cell cultures sampled at the exponential phase showed an OD_600_ value of about 2.5 for the control and 0.6 for the QDs treatment, whereas cultures harvested at the stationary phase showed an OD_600_ value of about 12.0 for the control and 4.5 for QDs treatment with and without nystatin.

### 3.2. Effect of CdS QDs on ROS Generation and Cell Integrity in S. cerevisiae

**Figure 2:**
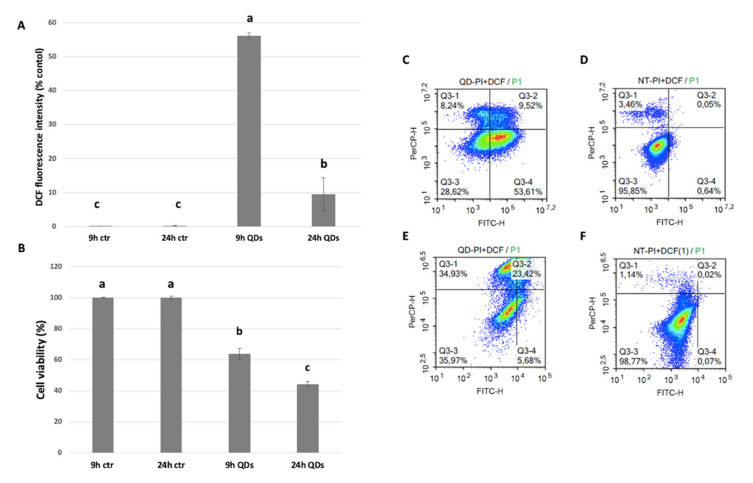
Flow cytometric measurements. The bars represent the average of 4 independent replicates. ANOVA was carried out followed by the Bonferroni post hoc test. Different letters indicate a statistic difference with *p* < 0.001: (**A**) changes in intracellular ROS; (**B**) cell viability evaluated by flow cytometry with propidium iodide. Yeast were stained with DCFHDA (2’-7’ Dichlorofluresceine diacetate) and PI (Propidium iodide) and detected by flow cytometry after 30 min incubation in the dark. The lateral axis represents the fluorescence of DCFH while the vertical axis indicates the PI intensity of detected cells. (**C**) Control for 9 h; (**D**) treatment for 9 h; (**E**) control for 24 h; (**F**) treatment for 24 h.

Flow cytometry analysis (Figure 2) showed that exposure for 9 h to CdS QDs led to an overproduction of ROS, while a significantly lower ROS production was observed after 24 h of treatment (CdS QDs 100 mg L^−1^). The results indicate that growth inhibition induced by the treatment was associated with oxidative stress and some cytotoxic effects already at 9 h. Figure 2A,C–F show the time-dependent changes in intracellular production of ROS compared to the untreated control.

Production of ROS is considered a major factor in QDs toxicity. The deleterious action of oxidative stress starts by causing oxidative damage to biomolecules and destroying their structure, which decreases cellular defences and ultimately leads to cell death, possibly by a mechanism similar to apoptosis [43]. Overall, our data suggest that QDs affect the expression levels of a number of proteins by inducing oxidative stress at both treatment times. It is possible to correlate the dysfunction in the glycolysis pathway, the downregulation of oxidative phosphorylation and also the increase in protein misfolding in the ER - all caused by QD treatment-with the production of ROS, which impairs the oxidative balance of the cells and becomes increasingly severe over time [44,45].

Figure 2B shows that after 9 h of CdS QDs treatment, the proportion of dead cells was 30% higher with respect to the control, whilst at 24 h the proportion of dead cells increased to 54%. These results confirmed that cell death increased with the exposure time and dose of CdS QDs [45,46].

### 3.3. Proteomic Response to CdS QDs Exposure

Qualitative and quantitative changes in the yeast proteome during CdS QDs treatments were obtained from the 2D-gel-based and gel-free iTRAQ approaches, respectively [47,48]. The less quantitative data from the 2D-PAGE analyses are presented in the Supplementary Materials (Figures S3–S6 and Table S1)  and can be benchmarked against data available in the literature and based on the same approach [49]. The next paragraphs focus on the more comprehensive and quantitatively robust iTRAQ analyses.

The time points for quantitative iTRAQ analysis were 9 and 24 h. This gel-free approach allowed processing more samples than 2D-PAGE. Therefore, proteome variations were analysed under all treatments and both time points. The iTRAQ approach enables quantification at the peptide level and direct protein mapping because both types of information originating from the same MS-MS spectra. In several other iTRAQ studies, about a thousand proteins were identified [50,51]. More than a thousand proteins were detected here within every single iTRAQ experiment on each biological replicate (Figures S7 and S8). 

The iTRAQ experiments corresponding to 9 h of treatment allowed the identification and quantification of 1129 (934 quantified) and 1055 (835) unique proteins from the two biological replicates BR1 and BR2, respectively. Of these, 849 (712) proteins were common to both biological replicates (Figure S7 and Supplementary Tables S2–S6).

**Figure 3:**
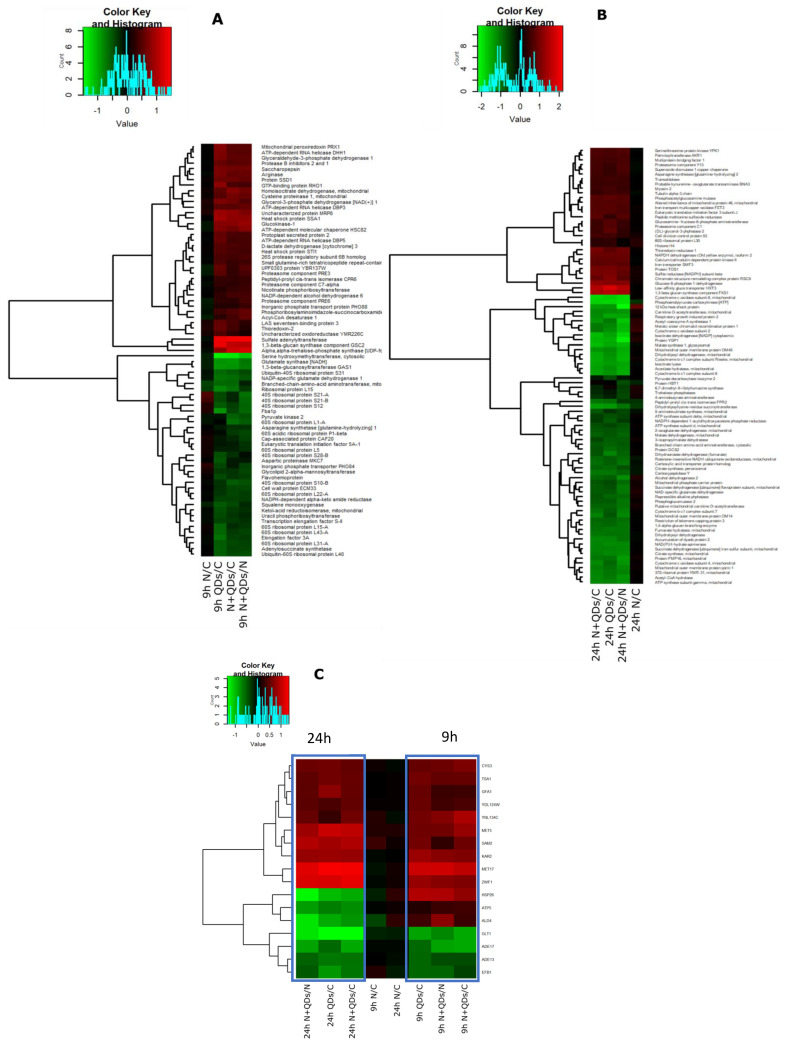
Heat map illustrating the relative abundance of the significantly enriched proteins (**A**) at 9 h; (**B**) at 24 h; (**C**) the common proteins between 9 and 24 h altered by treatment with CdS QDs found with iTRAQ.

The iTRAQ analysis revealed 97 proteins enriched in the yeast cells in response to the treatments with CdS QDs, with and without nystatin: (Figure S7) 56 of these proteins were identified by comparing the control (ctr) vs QDs treatment; 35 were identified from the comparison of ctr vs nystatin + QDs, seven by comparing ctr vs nystatin and 48 by comparing the nystatin vs nystatin + QDs samples. The CdS QD treatment altered the abundance level of 22 common proteins between ctr vs QDs, ctr vs nystatin + QDs and nystatin vs nystatin + QDs. Only two proteins from the comparison of ctr vs nystatin were included because the other five proteins were not common to any other treatment and therefore were not considered relevant. (Figure S9A). This confirms that the treatment with nystatin while favouring the uptake of CdS QDs, did not change substantially the proteome profile. Heatmaps based on protein classes were generated from these data (Figure 3). The most affected proteins were involved in oxidative stress. Of highest relevance to this process is the oxidoreductase category, including peroxiredoxin (Tsa1), glucose-6-phosphate 1-dehydrogenase (G6pd), thioredoxin-2 (Trx2) and glyceraldehyde-3-phosphate dehydrogenase 1 (Tdh1). These four proteins were up regulated during exposure with QDs (Figure 3A). The downregulated proteins were mostly ribosomal subunits (40S and 60S ribosomal proteins), some proteins from the glycolytic pathway such as Fba1p, some mitochondrial proteins such as mitochondrial branched-chain-amino acid amino transferase and keto-acid reductoisomerase and also serine hydroxymethyltransferase (Figure 3A). The lists of proteins identified in two biological replicates and proteins common to all datasets are shown in Table S6.

iTRAQ analysis of the 24 h samples allowed the identification of 943 (886 quantified), and 1346 (1080) unique proteins from the two biological replicates BR1 and BR2, respectively. Of these, 562 (505) proteins were common to the two biological replicates (Figure S8 and Supplementary Tables S7–11). The iTRAQ-based quantitative analysis revealed that the total number of proteins enriched in the yeast cells in response to all treatments with CdS QDs, with and without nystatin, was 109. Seventy-six of these proteins were identified by comparing ctr vs QDs, 76 by comparing ctr vs nystatin + QDs, three by comparing ctr vs nystatin and 89 from the difference between the nystatin vs nystatin + QDs samples (Figure S9B). Here again, the differences in the presence and absence of nystatin were minimal for each condition. The CdS QD treatment altered the expression level of 58 common proteins as judged from the comparison of ctr vs QDs, ctr vs nystatin + QDs, and nystatin vs nystatin + QDs. (Figure S9). The lists of proteins identified in two biological replicates and proteins common to all datasets are shown in Table S11. Notably, the proteins differentially expressed at 24 h present a different trend with respect to the 9 h treatment, i.e., the majority of the proteins upregulated at 9 h were downregulated at 24 h (Figure 3B). In particular, the following mitochondrial proteins involved in oxidative phosphorylation were downregulated: succinate dehydrogenase (ubiquinone) iron-sulphur subunit (Sdh2), cytochrome b-c1 complex subunit 6 (Qcr6), cytochrome b-c1 complex subunit 7 (Qcr7), cytochrome b-c1 complex subunit (Rip1, the Rieske protein), cytochrome c oxidase subunit 4 (Cox4), cytochrome c oxidase subunit 6 (Cox6), ATP synthase subunits, d, gamma, and delta (Atp16, Atp3 and Atp16) (Figure 8). The most upregulated proteins were 12kDa heat shock proteins, phosphoenolpyruvate carboxykinase and proteins involved in sulphur metabolism, such as sulphite reductase NADPH subunit beta (Figure 3B).

Further comparison between the 9 and 24 h times performed with iTRAQ revealed that seventeen proteins were common to both treatments (Figure 3C), which reflects dynamic readjustment of the proteome during the change in viability (decrease) and ROS production (increase). Ten proteins were up regulated at the two times of treatment: 78 kDa glucose-regulated protein homolog (Kar2), cystathionine gamma-lyase (Cys3), glucosamine-fructose-6-phosphate aminotransferase [isomerising] (Gfa1), glucose-6-phosphate 1-dehydrogenase (Zwf1), NADPH-dependent alpha-keto amide reductase (Ydl124w), peroxiredoxin (Tsa1), protein MET17, S-adenosylmethionine synthase 2 (Sam2), sulfite reductase [NADPH] subunit beta (Met5), uncharacterised protein YNL134C. Four proteins were downregulated at the two times of treatment: adenyluccinate lyase (Ade13), bifunctional purine synthesis protein ADE17, elongation factor 1-beta (Efb1), and glutamate synthase [NADH] (Glt1). Instead, three proteins, namely heat shock protein 26 (Hsp26), potassium-activated aldehyde dehydrogenase mitochondrial (Ald4), and ATP synthase subunit 5, mitochondrial (Atp5) were upregulated at 9 h and downregulated at 24 h. The first two are involved in protein processing in the endoplasmic reticulum, while the last is involved in oxidative phosphorylation (Figure 3C).

### 3.4. Ontology Analysis of the Identified Proteins

**Figure 4:**
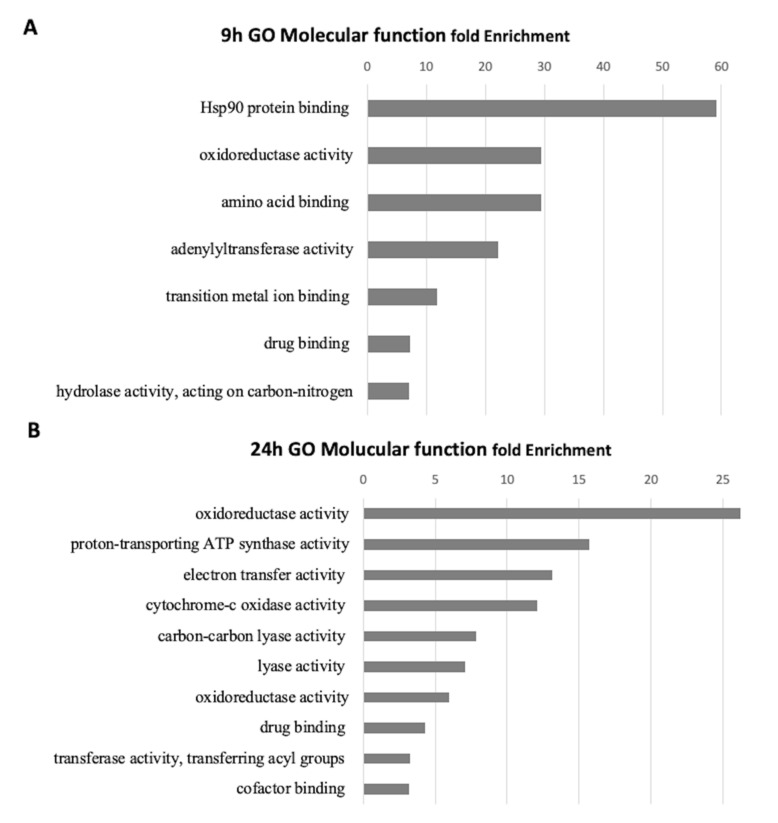
Gene ontology and enrichment analyses with fold enrichment = −log10 (Fisher’s exact *p*-value) for molecular function: (**A**) 9 h; (**B**) 24 h. Proteins obtained with iTRAQ.

Analysis using gene ontology (GO) groups proteins based on molecular functions, biological processes, and cellular components. GO enrichment analysis of differentially expressed protein altered by CdS QDs stress at 9 h and 24 h was performed using the PANTHER software. This annotation of proteins into different classes was instrumental in understanding their biological relevance. A total of 56 slim GO terms were significantly enriched (*p* < 0.05). PANTHER grouped all the enriched proteins at 9 h into seven groups based on their molecular functions: Hsp90 protein binding, oxidoreductase activity, amino acid binding adenylyltransferase activity, drug binding hydrolase activity, acting on carbon–nitrogen (Figure 4A).

The molecular GO functions for the 24 h samples were: oxidoreductase activity, proton transporting ATP synthase activity, electron transfer activity, cytochrome c oxidase activity, carbon-carbon lyase activity, lyase activity, oxidoreductase activity, drug binding, transferase activity, cofactor binding (Figure 4B). 

When the enriched proteins identified at 9 h were analysed on the basis of biological processes, they were organised in 16 groups, of which the major were: response to organic cyclic compounds, glutamate metabolic process, response to acid chemical, response to endogenous stimulus, branched-chain amino acid biosynthetic process, sterol biosynthetic process, nucleotide-sugar metabolic process, cytoplasmic mRNA processing body assembly, cell wall polysaccharide metabolic process, histone deacetylation, cellular response to oxygen-containing compounds (Figure S10A). 

The enriched proteins obtained for the 24 h treatment were subdivided into 15 groups, of which the more important were: mitochondrial electron transport, nucleotide-sugar metabolic process, glutamate metabolic process, reactive oxygen species metabolic process, tricarboxylic acid cycle, aerobic respiration, respiratory electron chain, ATP synthesis coupled proton transport oxidative phosphorylation (Figure S10B). 

The main GO cell component categories for the 9 h treatment were: cytoplasmic stress granule and cytosolic small ribosomal subunit. The samples recovered after 24 h treatment were enriched in mitochondrial respiratory chain complex III, proteosome core complex, alpha-subunit complex, cytochrome complex, oxidoreductase complex, respiratory chain complex, mitochondrial outer membrane (Figure S10C,D).

GO analysis of the differentially expressed proteins identified ‘oxidoreductase activity’ as the most perturbed biochemical functions in response to CdS QD exposure at 9 and 24 h, whilst the GO molecular functions that differ between the two times of exposure corresponded to classes of general protein binding at 9 h compared to electron transfer and cytochrome c activity at 24 h. 

Most affected proteins belonging to the oxidoreductase activity category were upregulated. These were: peroxiredoxin TSA1, peroxiredoxin PRX1 and superoxide dismutase 1 copper chaperone. Analysis of the significant biological processes common to the 24 and 9 h samples were: glutamate metabolic process, nucleotide-sugar metabolic process and reactive oxygen species metabolic process. These three categories are typical of stress response activities, but all the other categories were different. In fact, at 24 h most of the categories pertained to respiration and mitochondrial metabolic activities, whilst for the 9 h treatment consisted of categories consistent with a general stress response.

At 24 h, protein abundance analysis revealed that the majority of the GO classes were downregulated. Hence the data show that the response to the treatment with CdS QDs was time-dependent. In particular, two of the downregulated proteins at 24 h that belong to each of the aerobic respiration, cellular respiration and tricarboxylic acid (TCA) cycle classes were citrate synthases CIT1 and CIT2. In eukaryotes, the TCA cycle occurs in the mitochondrial matrix and plays a pivotal role in the utilisation of non-fermentable carbon sources via oxidative generation of reducing equivalents (NADH), driving aerobic respiration to yield ATP [52]. The TCA cycle is also an important source of biosynthetic building blocks, such as α-ketoglutarate, succinyl-CoA and oxaloacetate, which are required for the synthesis of glucose and amino acids [52]. Yeasts have multiple citrate synthase genes (*CIT1*, *CIT2*, and *CIT3*), but it is not clear how they differ in function or if any of them encode a specific methylcitrate synthase. The products of the *CIT1* and *CIT3* genes have been shown to be mitochondrial proteins, whereas that of the *CIT2* gene is clearly peroxisomal [53]. The foregoing molecular function and biological processes mostly linked to mitochondrial function and structure represent the “core response” to CdS QDs. These data are in keeping with other results obtained from simple eukaryotic organisms and human cell lines [20,21,23]. From a physiological and molecular point of view, it has been demonstrated that ENMs increase ROS production by interacting negatively with all cell compartments, in particular by affecting cell membranes and the mitochondria and, consequently, the levels of energy production and cellular respiration [20]. The relationship between ROS production and inhibition of respiration has been reported in the literature. For example, Fe_3_O_4_ nanoparticles have an inhibitory effect on yeast growth. The inhibition is attributed to their interaction with the mitochondria, leading to disruption of the mitochondrial respiratory chain complex IV, and consequent attenuation of ATP production [54]. In addition, it has been found that NiO NPs inhibit metabolic activity, induce intracellular accumulation of ROS, and provoke cell death in *S. cerevisiae* [55].

### 3.5. Pathway Analysis of the Identified Proteins

**Figure 5:**
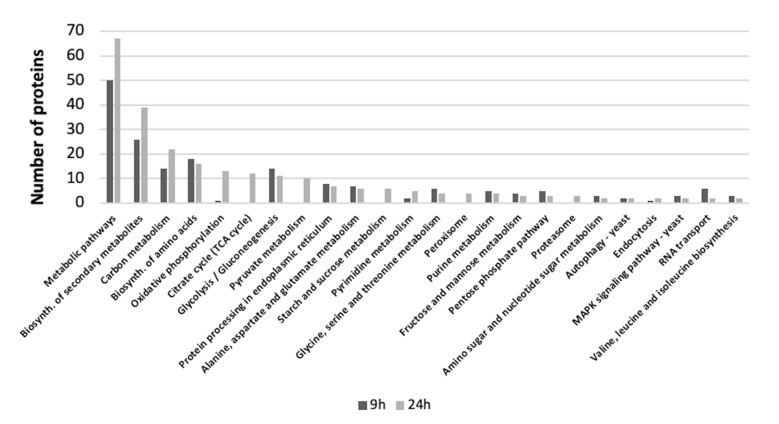
Pathway analysis: Distribution of responsive proteins in yeast at 9 and 24 h, according to the KEGG pathway classification. Black bars are for the proteins found in the 9 h treatment, grey bars represent the proteins found in the 24 h treatment. Proteins obtained with iTRAQ.

Metabolic pathway analysis was performed by submitting the gene IDs of the proteins identified with iTRAQ to the KEGG server (http://www.kegg.jp) for *S. cerevisiae* to identify the pathways that were represented more frequently. At 9 h, the main pathway classes were: general metabolic pathway, biosynthesis of secondary metabolites, biosynthesis of amino acids, glycolysis and gluconeogenesis, protein biosynthesis, carbon metabolism, and protein processing in the endoplasmic reticulum (ER) (Figure 5).

At 24 h the main pathway classes were: general metabolic pathway, biosynthesis of secondary metabolites, oxidative phosphorylation, TCA cycle, glycolysis and gluconeogenesis, pyruvate metabolism, protein biosynthesis, carbon metabolism, and protein processing in the endoplasmic reticulum (ER) (Figure 5).

**Figure 6:**
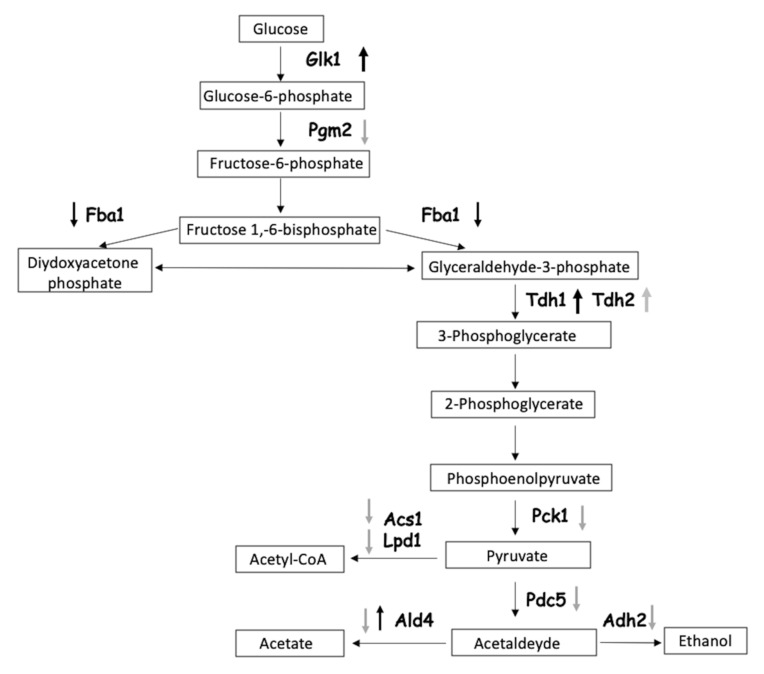
Glycolysis and gluconeogenesis pathway. Black arrows are for the proteins found, with iTRAQ, after 9 h treatment, grey arrows are for the proteins found after 24 h treatment. Arrows pointing up indicate upregulated proteins, arrows pointing down indicate downregulated proteins.

Of particular interest was the pathway “glycolysis and gluconeogenesis”, common to the two treatment times (Figure 6), which included four proteins identified at 9 h, and 9 at 24 h. At 9 h of treatment, three enzymes associated to the glycolysis pathway were upregulated: glyceraldehyde-3-phosphate dehydrogenase 1 (Tdh1), glucokinase-1 (Glk1), and mitochondrial potassium-activated aldehyde dehydrogenase (Ald4). One enzyme was downregulated: fructose-bisphosphate aldolase (Fba1). At 24 h of treatment, the majority of the enzymes associated to the glycolytic pathway were downregulated: acetyl-coenzyme A synthetase 1 (Acs1), dihydrolipoyl dehydrogenase (Ldp1), pyruvate decarboxylase isozyme 5 (Pdc5), mitochondrial potassium-activated aldehyde dehydrogenase (Ald4), and NADP-dependent alcohol dehydrogenase 2 (Adh2). Only one enzyme, i.e., glyceraldehyde-3-phosphate dehydrogenase 2 (Tdh2), was detected at levels higher than the control.

The only common enzyme to the 9 and 24 h exposure times was Ald4, the level of which initially increased at 9 h and decreased at 24 h. As reported earlier, ENMs treatment inhibits the glycolytic pathway and stimulate fermentation [56]. Horstmann et al., (2019) suggested that sugar transport genes and sugar-utilising enzyme genes are simultaneously affected by the presence of Cd-QDs [57]. The two isoforms of GAPDH (Tdh1, Tdh2) were found to be upregulated for both treatment times. GAPDH is a glycolytic enzyme involved in glucose degradation and energy yield. It catalyses the sixth step of glycolysis, i.e., the conversion of glyceraldehyde-3-phosphate to 1,3 bis-phosphoglycerate, but also displays non-glycolytic activity in certain subcellular locations. In vitro inhibition studies of GAPDH in the presence of QDs suggest that binding of QDs to the enzyme molecules slows down the rate of the reaction catalysed by the enzyme, suggesting that QDs may act as enzyme inhibitors [58]. When human cancer cells are exposed to QDs, the loss of cellular GAPDH activity causes a metabolic perturbation during glycolysis, and the inhibition of GAPDH leads to the decrease of glycolytic rates. This suggests a possible mechanism of change in energy production from the glycolytic pathway to fermentation during QD-mediated cellular injury. This process may lead eventually to cell dysfunction and death [58].

Proteins leading to the Krebs cycle (Acs1, Lpd1, Ald4) or to fermentation (Adh2, Pdc5) were differentially expressed during treatment with CdS QDs at both time points (Figure 6). Pdc1 is the most prevalent form of the three yeast pyruvate decarboxylases which are involved both in the anaerobic fermentation of pyruvate to acetaldehyde and in amino acid catabolism. Pdc1, together with Tdh2 and Tdh3, was found among the proteins that constitute the hard corona in yeast during CdS QDs treatments, with a specific role in determining the toxicity of these ENMs [59].

**Figure 7:**
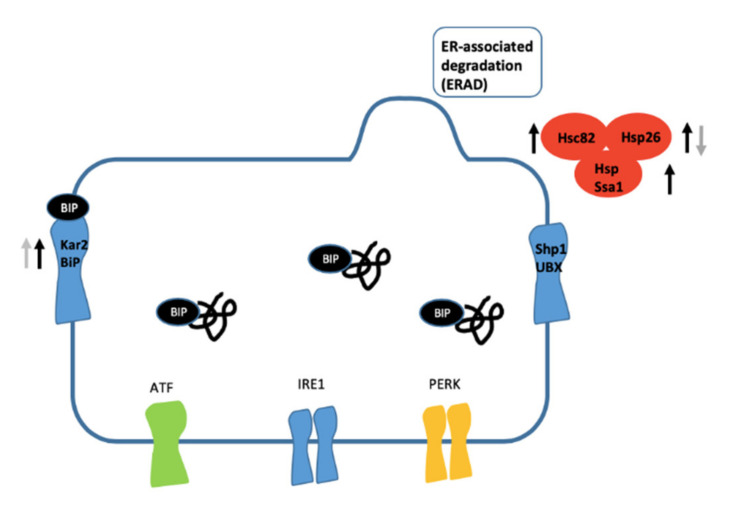
Protein processing in the Endoplasmic Reticulum (ER) pathway. Black arrows are for the proteins found with iTRAQ after 9 h treatment, grey arrows are for the proteins found after 24 h treatment. Arrows pointing up indicate upregulated proteins, arrows pointing down indicate downregulated proteins.

Another pathway of particular interest is “protein processing in ER”, which includes four proteins altered at 9 h (four downregulated) and four at 24 h (one protein with reduced levels and one with increased levels) (Figure 7). Two common enzymes were found to be altered at the two times of treatment:78 kDa glucose-regulated protein homolog (Kar2) and heat shock protein 26 (Hsp 26). Kar2 was present at higher levels at both 9 and 24 h. Contrarily, heat shock protein 26 (Hsp 26) was present at higher levels at 9 h and lower levels at 24 h, while heat shock protein Ssa1 was present at higher levels at 9 h. These two enzymes are ribosome-associated members of the Hsp70 family participating in the folding of newly-synthesised polypeptides [60]. Hsc82, a member of the Hsp90 family, was upregulated at 9 h and acts to promote the maturation, structural maintenance and regulation of proteins involved in cell cycle control, ribosome stability and signal transduction [61]. Hsp90 proteins operate in a number of signalling pathways which are altered during exposure to metal ENMs [62]. It was shown that Hsc82 is one of the main hubs in CdS QDs sensitivity [23] and that it is one of the hard corona proteins for CdS QDs in yeast [59]. The results obtained by Wei et al. (2017) on human cancer cells suggest that some ENMs are capable of inducing autophagy and affecting the ER [63]. Other authors reported that internalised silica nanoparticles (Si-NPs) may accumulate in lysosomes, resulting in lysosomal dysfunction in HeLa cells [64]. Similarly, Si-NPs accumulation in the ER indicates an effect on ER structure, through mechanisms still unknown. Any damage to the ER is closely connected to cell autophagy, one of the principal cell death mechanisms triggered by ENMs. The acute toxicity of ZnO NPs to *Daphnia pulex* evidenced by proteomic results showed that some processes, such as protein synthesis and translocation across the ER, were inhibited to reduce the stress associated to protein misfolding [65]. More recent evidence support that the induction of autophagy or apoptosis in two cell types (human hepatocellular carcinoma cells (HepG2) and macrophages (THP1) in response to the treatment with CdS QDs is not only cell-type specific but also dependent on the form of Cd [66].

**Figure 8:**
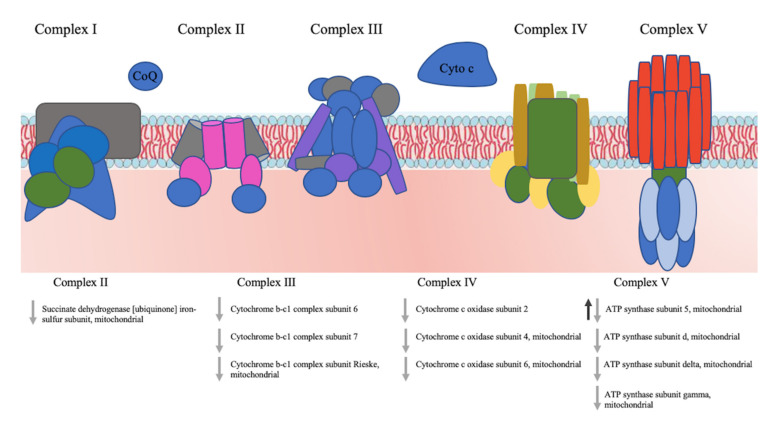
Oxidative phosphorylation pathway. Black arrows are for the proteins found with iTRAQ after 9 h treatment, grey arrows are for the proteins found after 24 h treatment. Arrows pointing up indicate upregulated proteins, arrows pointing down indicate downregulated proteins.

The majority of the altered proteins involved in “oxidative phosphorylation” are from the 24 h treatment with CdS QDs. Nine of these proteins were downregulated, suggesting that energy production was significantly lessened. These proteins are the mitochondrial succinate dehydrogenase (ubiquinone) iron-sulphur subunit (Sdh2), cytochrome b-c1 complex subunit 6 (Qcr6), the mitochondrial cytochrome b-c1 complex subunit 7 (Qcr7), cytochrome b-c1 complex subunit (Rip1, the Rieske protein), cytochrome c oxidase subunit 4 (Cox4), the mitochondrial cytochrome c oxidase subunit 6 (Cox6), the mitochondrial ATP synthase subunits 5, d, gamma, and delta (Atp5, Atp16, Atp3 and Atp16), and cytochrome c oxidase subunit 2 (Cox2). The only protein altered at 9h was Atp5 and it was up regulated. It appears that after 24 h of treatment most of the mitochondrial proteins had reduced activity, causing a slow-down in oxidative phosphorylation and ATP production (Figure 8). The proteins most affected by the CdS-QDs are components of mitochondrial respiration complexes III, IV and V. Mitochondria are a significant organelle in QD-induced toxicity [67,68]. It has been shown that CdS QDs damage mitochondrial functionality and reduce respiration activity in yeast [24], plants [22], and human cells [21]. Damage to mitochondrial functions and structure caused by several types of metal-ENMs has been reported in mollusc bivalve and mouse cells [69,70]. Interestingly all the proteins of the ATP synthase complex were downregulated, which indicates a reduction in energy produced through oxidative phosphorylation, and connects with a general downregulation of the enzymes involved in the glycolytic pathway.

In summary, the upregulation of the fermentation process reflects a metabolic change to lactate or acetate production to provide enough energy for survival and bypass the aerobic metabolism. Moreover, acetate is also regarded as an expedient source of energy for stressed cells [71]. These observations are consistent with the reports in which silver nanoparticles caused oxidative stress and defects in mitochondrial and endoplasmic reticulum (ER) enzymes [72,73]. In aerobic metabolism, ROS are natural by-products, but an excess of ROS can chemically modify proteins and lipids by peroxidation, thus leading to damage to vital organelles such as mitochondria, the ER, and lysosomes [74,75] (Figure 2, Figure 6, Figure 7 and Figure 8).

### 3.6. Inhibition of GAPDH Activity by CdS QDs

**Figure 9:**
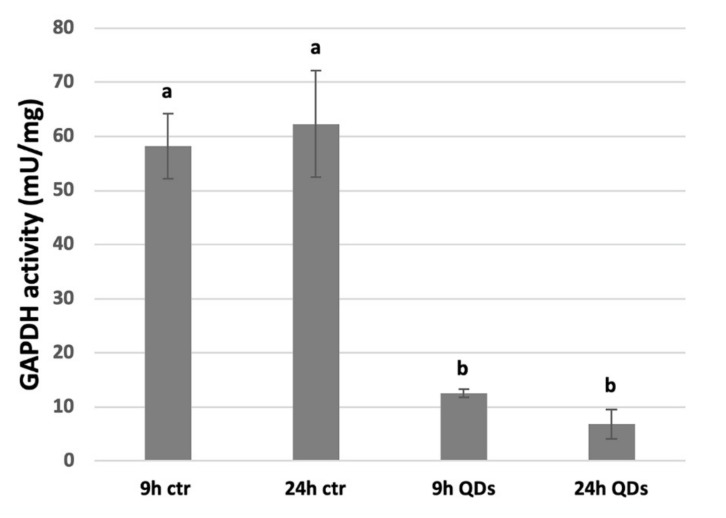
GAPDH (glyceraldehyde-3-phosphate dehydrogenase) activity. ANOVA average of three replicates followed by Bonferroni post hoc test. Different letters indicate a statistic difference with *p* < 0.001.

Figure 8 shows that at both 9 and 24 h the activity of GAPDH in yeast cells treated with 100 mg L^−1^ of CdS QDs was significantly lower than in the untreated samples (Figure 9). Though not highly significant, the activity of GAPDH at 9 h was higher than at 24 h. Overall the CdS QDs treatment at both time points inhibits the glycolytic process at the level of the enzyme GAPDH, as suggested by the proteomic approach (Figure 6). CdS QD treatment consistently altered GAPDH abundance and decreased GAPDH activity. in vitro experiments in the BY4742 yeast strain on hard corona proteins demonstrated a strong dose-dependent reduction of the enzyme activity upon CdS QDs treatment [59]. The reduction of GAPDH activity by CdS QDs could be explained by CdS QD oxidation of the GAPDH active site (cysteine 152), which is known to lower GAPDH activity and reduce the accessibility to substrates such as glyceraldehyde-3-phosphate [58,59]. ENPs can induce unfolding and reduced activity of the identified proteins, as observed in the case of GAPDH isoforms, but CdS QD binding to hard corona proteins could also mediate non-specific interactions with other cellular components [58,59].

### 3.7. Robustness of Markers Identification Using Multiomic Approaches

**Figure 10:**
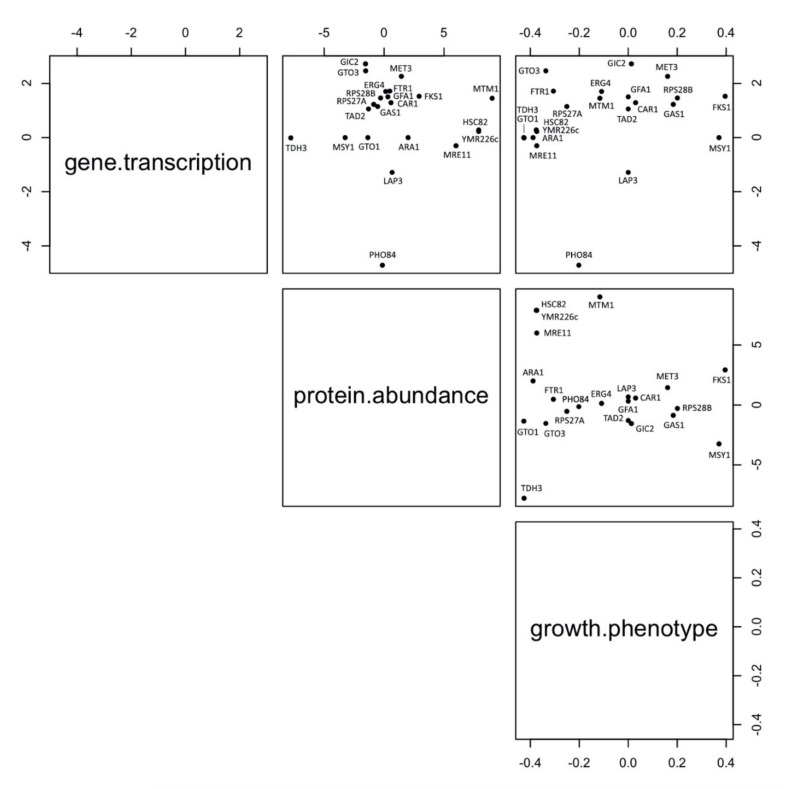
Scatterplot matrix of the independent variables: transcriptome, proteome, and phenome. Three-dimensional scatterplot representing the correlation among gene expression, protein abundance and growth phenotype. Phenomics data are taken from Marmiroli et al., 2016, transcriptomics data from Pagano et al., 2019.

The proteins that were up- or downregulated following CdS QDs treatment were assessed against other omics markers identified using transcriptomics and phenomics [20,23,24]. Figure 10 shows the levels of correlation between proteomics/transcriptomics, phenomics/transcriptomics and proteomics/phenomics markers. These data were obtained by comparing 284 significant proteins against more than 5000 haploid deletion mutants and the whole set of transcripts obtained with a yeast microarray platform [23]. The correspondences, both symmetric (++/--) and antisymmetric (+-/-+) consisted of a small percentage of the compared elements, i.e., 22 proteins, 14 transcripts, and eight mutants which responded as up/downregulated and/or sensitive/tolerant to the treatment with CdS QDs. It is well known that the correspondence between proteomics and transcriptomics is typically low [76]. The molecular markers that showed this level of correlation in the three comparisons are considered robust enough to be candidates as omics exposure markers. The functions which are most implicated are mitochondrial structure and function, glycolysis cycle and protein processing in the ER. Across the proteins, transcripts and growth phenotypes, the only common element is FKS1, which encodes the catalytic subunit of the yeast 1,3-β-d-glucan synthase, relevant in the building of yeast cell wall and consequently in the front line of cell exposure to external materials.

## 4. Conclusions

The complexity of biological systems often makes it difficult to study their internal interactions. The choice of *S. cerevisiae* for this study was motivated by the knowledge base available on yeast genetics and omics, including the characterisation of the entire proteome and genome and the existence of a full set of deletion mutants which cover the entire genome. This approach facilitates the identification of ‘leads’ to be addressed in higher organisms [29]. To explore the mechanism of ENMs toxicity, new approaches utilising HTS techniques have been advocated. In this study, comparative proteomics analysis with iTRAQ revealed some of the final effectors of the responses to CdS QDs in yeast after 9 and 24 h exposure. Key proteins from some of the major metabolic pathways critical to the survival of yeast and other organisms were identified as altered by the treatment. 

The most significant adverse outcome pathways (AOPs) influenced by CdS QDs were glycolysis, the oxidative phosphorylation chain, and ubiquitination and trafficking in the ER. In addition, it has been demonstrated that CdS QDs generate ROS at both time points, giving rise to increased oxidative damage. These findings will also assist in the establishment of environmental risks associated with the disposal of CdS QD and their interactions with ecosystems, showing how nanotechnology can contribute to the safe use of ENMs [14].

Correlation between the molecular markers found in this and other studies [20,23,24] makes their identification reliable and robust. There are few markers in common among proteomics, transcriptomics and phenomics data, but the recurrence of these within the different tests is significant. As a fact, proteomic markers are “early markers” of cellular exposure, whereas phenomic markers are “global markers” at the organismal level. 

## Supplementary Materials

The following are available online at https://www.mdpi.com/2079-4991/10/6/1214/s1. **Supplementary file 1**: S.1; S.2. Supplementary methods related to nanoparticles synthesis and characterisation. S.3; S.4; Supplementary methods related to 2D-PAGE. S.5. Identification of differentially expressed proteins with 2D-PAGE. Figure S1. HRTEM image of ligand-free QDs assembly and X-ray diffraction pattern. Figure S2. (A) ESEM image of the CdS QDs agglomerates. (B) X-ray spectra corresponding to the red rectangle in figure S2A. Figure S3. 2D-PAGE for the 9 h treatments. Figure S4. Venn diagrams for the differentially regulated proteins in all treatment conditions 9 h treated with CdS QDs with and without nystatin obtained with 2D-PAGE. Figure S5. Heat map of proteins altered at 9 h under treatment with CdS QDs found with 2D-PAGE. Figure S6. Gene ontology and enrichment analyses with fold enrichment = −log10 (Fisher’s exact *p*-value) for (A) molecular function; (B) cell component; (C) biological process obtained after 9 h treatment with CdS QDs utilising 2D-PAGE. Figure S7. Venn diagram showing the number of unique proteins identified and quantified from the iTRAQ analysis of two biological replicates (BR1 and BR2) for the 9 h treatment corresponding to the four developmental stages. Figure S8. Venn diagram showing the number of unique proteins identified and quantified from the iTRAQ analysis of two biological replicates (BR1 and BR2) for the 24 h treatment corresponding to the four developmental stages. Figure S9. Venn diagrams for the differentially regulated proteins in all treatment conditions. (A) 9 h iTRAQ; (B) 24 h iTRAQ. Figure S10. Gene ontology slim for iTRAQ, enrichment analyses with fold enrichment = −log10 (Fisher’s exact *p*-value) for: (A) 9 h biological process; (B) 24 h treatments biological process; (C) 9 h cell component; (D) 24 h cell component. **Supplementary file 2**: Table S1. MALDI-TOF/TOF data associated with differentially expressed proteins identified by 2D-PAGE at 9 h. Table S2–6 list of all unique proteins, identified and quantified by iTRAQ experiments at 9 h from the two biological replicates (BR1 and BR2). Table S7–11: List of all unique proteins, identified and quantified by iTRAQ experiments at 24 h from the two biological replicates (BR1 and BR2).
