# Supplementary material for: Proteomic Analysis Identifies Markers of Exposure to Cadmium Sulphide Quantum Dots (CdS QDs)
Source: Nanomaterials (Basel). 2020 Jun 22;10(6):1214. doi: 10.3390/nano10061214 (PMC7353101; doi:10.3390/nano10061214)
Supplement: Supplementary file 1 [file nanomaterials-10-01214-s001.zip › nanomaterials-780869-supplementary-Final check/nanomaterials-780869-supplementary-Final check.pdf]

Supplementary file 1.

Proteomic Analysis Identifies Markers of Exposure to Cadmium Sulphide Quantum Dots (CdS QDs)

Valentina Gallo [1], Vaibhav Srivastava [2], Vincent Bulone [2,3], Andrea Zappettini [4], Marco Villani [4], Nelson Marmiroli [1,5] and Marta Marmiroli [1,*]

1 Department of Chemistry, Life Sciences and Environmental Sustainability, University of Parma, 4 Parma Stockholm, Italy; valentina.gallo@unipr.it (V.G.); nelson.marmiroli@unipr.it (N.M.)
2 Royal Institute of Technology (KTH), Department of Chemistry, Division of Glycoscience, School of Engineering Sciences in Chemistry, Biotechnology and Health, AlbaNova University Center, SE-106 91 Stockholm, Sweden; vasri@kth.se (V.S.); bulone@kth.se (V.B.)
3 ARC Centre of Excellence in Plant Cell Walls and School of Agriculture, Food and Wine, The University of Adelaide, 5064 Urbrae, South Australia, Australia
4 Department of Nanomaterials, Institute of Materials for Electronics and Magnetism (IMEM)Department of Nanomaterials, National Research Council (CNR), 6 Parma, Italy; andrea.zappettini@imem.cnr.it (A.Z.); marco.villani@imem.cnr.it (M.V.)
5 The Italian National Interuniversity Consortium for Environmental Sciences (CINSA), 43124 Parma, Italy
* Correspondence: marta.marmiroli@unipr.it; Tel: +39-0521-905698

List of Supplementary material:

S.1; S.2. Supplementary methods related to nanoparticles synthesis and characterisation
S.3; S.4; Supplementary methods related to 2D-PAGE
S.5. Identification of differentially expressed proteins with 2D-PAGE
Figure S1. HRTEM image of ligand-free QDs assembly and X-ray diffraction pattern
Figure S2. (A) ESEM image of the CdS QDs agglomerates. (B) X-ray spectra corresponding to the red rectangle in figure S2A.
Figure S3. 2D-PAGE for the 9 h treatments.
Figure S4. Venn diagrams for the differentially regulate proteins in all treatment conditions 9 h treated with CdS QDs with and without nystatin obtained with 2D-PAGE.
Figure S5 Heat map of proteins altered at 9 h under treatment with CdS QDs found with 2D-PAGE
Figure S6. Gene ontology and enrichment analyses with fold enrichment = -log10 (Fisher's exact *p*-value) for A) molecular function; B) cell component; C) biological process obtained after 9 h treatment with CdSQDS utilising 2D-PAGE
Figure S7. Venn diagram showing the number of unique proteins identified and quantified from the iTRAQ analysis of two biological replicates (BR1 and BR2) for the 9 h treatment corresponding to the four developmental stages.
Figure S8. Venn diagram showing the number of unique proteins identified and quantified from the iTRAQ analysis of two biological replicates (BR1 and BR2) for the 24 h treatment corresponding to the four developmental stages.
Figure S9. Venn diagrams for the differentially regulate proteins in all treatment conditions. A) 9 h iTRAQ; B) 24 h iTRAQ.)
Figure S10. Gene ontology slim for iTRAQ, enrichment analyses with fold enrichment = -log10 (Fisher's exact *p*-value) for: A) 9 h biological process; B) 24 h treatments biological process; C) 9 h cell component; D) 24 h cell component.

S.1. CdS QDs Synthesis and Characterization

Uncoated Cadmium Sulfide Quantum Dots (CdS QDs) were synthesised by IMEM-CNR (Parma, Italy), following the method of Villani *et al.* (2012) [1]. The CdS QDs were characterised in deionised water by transmission electron microscopy (TEM) (Hitachi HT7700, Hitachi High Technologies America, Pleasanton, CA) [2]. Average static diameter was 5 nm, and the crystal

structure was that of wurtzite (ZnS) with approximately 78% Cd. Average particle size (dh) of the aggregates (measured with DLS) and zeta potential (ζ) in ddH_2O were estimated in deionized water at 178.7 nm and +15.0 mV, respectively; in YPD the values were 545 nm and −11 mV respectively (Zetasizer Nano Series ZS90, Malvern Instruments, Malvern, UK). Additional particle characterization data is provided in Figures S1–S2.

S.2. Synthesis and Characterization of CdS QDs

Cadmium acetate (99%), N, N-dimethyl formamide (99%) and thiourea (99.5%) were all purchased from Sigma Aldrich (Saint Louis, MO, USA) and used without further purification. The method used to synthesise CdS QDs followed Villani et al. (2012), and the synthesis was performed by IMEM-CNR (Parma, Italy). X-ray diffraction was performed using an ARL-X'Tra device (Thermo Fisher Scientific, 81 Wyman Street, Waltham, MA USA) [1]. A field emission high resolution (Scherzer resolution of ~0.19 nm) JEM-2200 FS transmission electron microscope (JEOL ltd., 3-1-2 Musashino, Akishima, Tokyo, JAPAN) operating at 200 kV, was used to examine the structure of the CdS QDs. The diffraction pattern and HRTEM investigation of the CdS QDs are reported in Figure S1A, B, C.

An ESEM Quanta 250FEG, FEI with Bruker QUANTAX EDS XFlash® 6T detector series and ESPRIT 2 analytical methods interface (FEI company, 5350 NE Dawson Creek Drive Hillsboro, Oregon 97124 USA, Bruker, Am Studio 2D, 12489 Berlin, Germany) was utilised to determine CdS quantum dots group morphology and elemental content. Single drops of 1 mL containing 80 mg/L of CdS Quantum dots were left to dry on SEM stub covered with carbon tape in a protected environment. Seven stubs were analysed during one round of experiments. Working parameters for SE imaging e X-ray spectra acquisition were as follows. Pressure: 70 Pa, working distance: 9.9 mm, acceleration voltage: 20KeV. Figure S2A, B represents CdS QDs at different magnifications, along with their EDX spectra [3].

S.3. 2D-PAGE Separation and Trypsin Digestion

For protein separation in the first dimension, 250 μg of each sample was loaded onto 11-cm ReadyStrip pH 4–7 IPG strips (BioRad, USA) which had been rehydrated overnight with 250 μL IEF buffer containing the sample. Proteins were focused using a PROTEAN® i12™ IEF System (BioRad, USA) and by successively applying different voltages to the strip: 250 V (60 min), 1 kV (60 min), and 8 kV (5 h) for a total of 35 kV h^{-1}. After isoelectric focusing, the strips were successively incubated for 15 min in 3 mL of reducing buffer containing 2% w/v DTT, 6 M Urea, 0.375 M Tris-HCl (pH 8.8), 20% w/v glycerol, 2% w/v SDS, and for 15 min in 3 mL alkylating buffer containing 2.5% w/v iodoacetamide, 6 M urea, 0.375 M Tris-HCl (pH 8.8), 2% w/v glycerol. The second dimension (SDS-PAGE) was performed using a CriterionTM Dodeca™ cell (BioRad, USA) and 12% Criterion™ XT Bis-Tris gels (BioRad, USA) in 1 M MOPS (3-(N-morpholino)-propanesulfonic acid) buffer containing 1 M Tris, 20 mM EDTA and 2% w/v SDS. The proteins were stained with QC Colloidal Coomassie G-250 (BioRad, USA) and gel images were recorded using a ChemiDocTM Imaging System (BioRad, USA). Image analysis was performed using the PDQuest software from BioRad (USA). Three biological replicates were used for each of the four samples. 2D-gel images were scanned using the ChemidocMP Imaging System (BioRad) and the images were processed and analysed using the PDQuest v8.0.1 software (BioRad) and checked manually. Spot densities were normalised by local regression and subsequently against the whole gel densities. The relative density of each spot was averaged for three replicate gels and Student's t-test analysis ($p < 0.05$) was performed to determine statistically significant differences in protein abundance. Statistically relevant spots were successively excised from the gels using an EXQuest Spot Cutter (BioRad), destained by soaking the pieces of acrylamide for 30 min in a 1:1 solution of 100 mM ammonium bicarbonate and acetonitrile, and the proteins were hydrolysed with trypsin following the Shevchenko et al. (2006) protocol [4].

S.4. Proteins Identification

The solutions containing the tryptic peptides were desalted and concentrated to a final volume of 4 μL using a Zip-Tip C18 matrix according to the manufacturer's instructions (Millipore Corporation, Billerica, MA, USA), then dispersed into a α-cyano-4-hydroxycinnamic acid (4-HCCA) matrix prepared by dissolving 4-HCCA in 50% acetonitrile/0.05% trifluoroacetic acid and spotting on a MALDI plate. The samples were subjected to mass spectrometry analysis using a 4800 MALDI-TOF/TOFTM MS analyser (Applied Biosystems, Foster City, CA, USA). Peptide mass spectra were acquired in reflectron mode (500–4000 m/z range) and analysed using the mMass v5.5 open source software (www.mmass.org). For each feature, a peak list was created and manually checked for the presence of signals from the matrix complex, trypsin and human keratin peptides. The main parameters were set as follows: digestion enzyme, trypsin with one missed cleavage; mass type, monoisotopic; 100 ppm peptide tolerance. Cysteine carbamidomethylation and methionine oxidation were set as fixed and variable modifications, respectively.

S.5. Identification of Differentially Expressed Proteins with 2D-PAGE

For the yeast cells exposed to CdS QDs for 9 h, with and without nystatin, the 2D-gel electrophoresis (2D-PAGE) approach allowed the visualisation of around 900 spots for each sample. Subsequent MALDI-TOF MS-MS analysis allowed the identification of about 270 spots (Figure S3). Within the former group, 100 spots varied in intensity in response to the treatments: 81 of these differed from the control (ctr, not treated) vs QDs; 78 were different between ctr vs nystatin + QDs; four differed between ctr vs nystatin; and 72 differed between the nystatin vs nystatin + QDs samples (Figure S4). The CdS QDs treatment, with and without nystatin, altered the expression level of 56 common proteins as found by comparing ctr vs QDs; ctr vs nystatin + QDs; and nystatin vs nystatin + QDs (Figure S4). At 9 h, there is a balance in the number of the altered proteins between up and downregulated (Figure S5). The identities of the protein spots, whose abundance was differentially altered with a *p*-value of ≤0.05 is presented in Table S1.

At 9 h, 2D- PAGE results complement the iTRAQ results with only a small overlapping of four differentially modulated proteins (Figure S5). The complementary nature of these methods was highly useful: proteins identified with 2D-PAGE and iTRAQ differ substantially as shown in Figure S6 and in Table 4, but the combined use of different techniques uncovers a higher proportion of the proteome of an organism [5]. We pooled together all the proteins identified for the 9 h treatments obtained with both methods, and compared them with those obtained for the 24 h treatment resulting from the iTRAQ method. Four proteins were in common between the two methods at both 9 h and 24 h: ATP-dependent molecular chaperone Hsc82, uncharacterised oxidoreductase YMR226C, fructose-bisphosphate aldolase (Fba1), and homocysteine/cysteine synthase (Met17) (Figure S6).

PANTHER grouped all the enriched proteins at 9h with 2D-GE into ten groups based on their molecular functions. The major class were: RNA polymerase activity and DNA binding, several carbohydrate class activity and different class with binding activity. (Figure S7A). The GO cell component categories were PcG protein complex and cytosolic large ribosome subunit (Figure S7B). The enriched proteins identified at 9h with 2D-PAGE were analysed on the basis of biological processes, they were organized in fifteen groups, of which the major were: mRNA stabilization, ubiquitination process, glycolytic process and monosaccharide metabolic process (Figure S7C).

References

1. Pasquali, F., Agrimonti, C., Pagano, L., Zappettini, A., Villani, M., Marmiroli, M. White, J. C., Marmiroli, N. Nucleo-mitochondrial interaction of yeast in response to cadmium sulfide quantum dot exposure. *J. Hazard. Mater.* **2017**, *324*, 744–752.
2. Villani, M., Calestani, D., Lazzarini, L., Zanotti, L., Mosca R., Zappettini, A. Extended functionality of ZnO nanotetrapods by solution-based coupling with CdS nanoparticles. *J. Mater. Chem.* **2012**, *22*, 5694.
3. Marmiroli, M.; Mussi, F; Pagano, L; Imperiale, D.; Lencioni, G.; Villani, M; Zappettini, A.; White, J. C.; Marmiroli, N. Cadmium sulfide quantum dots impact *Arabidopsis thaliana* physiology and morphology. *Chemosphere* **2020**, *240*, 124856.

4. Shevchenko A, Tomas H, Havlis J, Olsen JV, Mann M. In-gel digestion for mass spectrometric characterization of proteins and proteomes. Nat. Protoc. **2006**; 1: 2856– 68760.
5. Pütz, S.M; Boehm, A.M.; Stiewe, T.; Sickmann, A. iTRAQ Analysis of a cell culture model for malignant transformation, including comparison with 2D-PAGE and SILAC. *J. Proteome Res.* **2012**, *11*, 2140−2153.

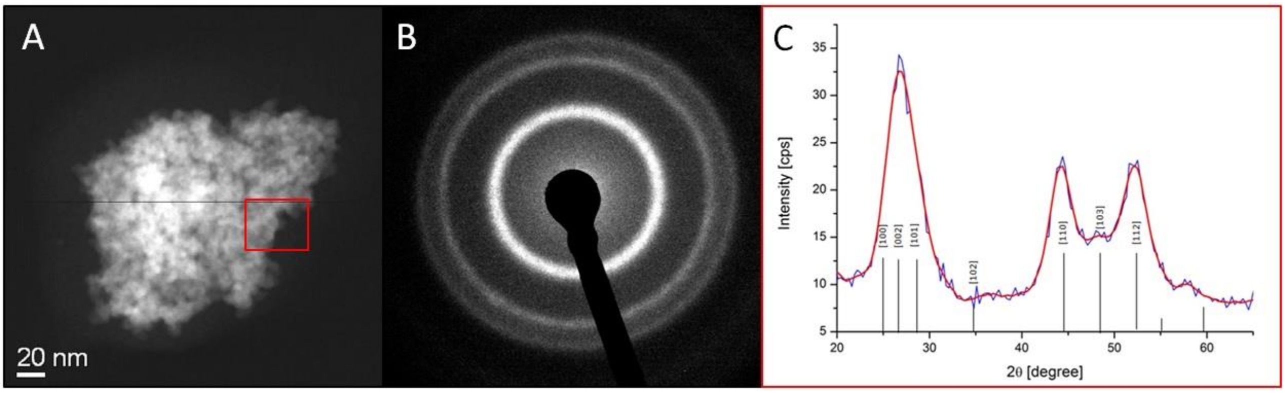

Figure 1. HRTEM image of ligand-free QDs assembly. (A) A CdS QDs aggregate upon solvent evaporation. (B) Fourier transform analysis of the whole HRTEM image. (C) X-ray diffraction pattern. Diffraction pattern obtained from the red rectangle: the diffraction rings related to the lower diffraction index are observable related to the hexagonal structure of the CdS QDs. From inside to outside are observable the triplet 100,002, 101; the doublet 110 e 103; and the singlet112. According to the diffraction pattern the estimated dimensions of the QDs are between 4 and 5 nm.

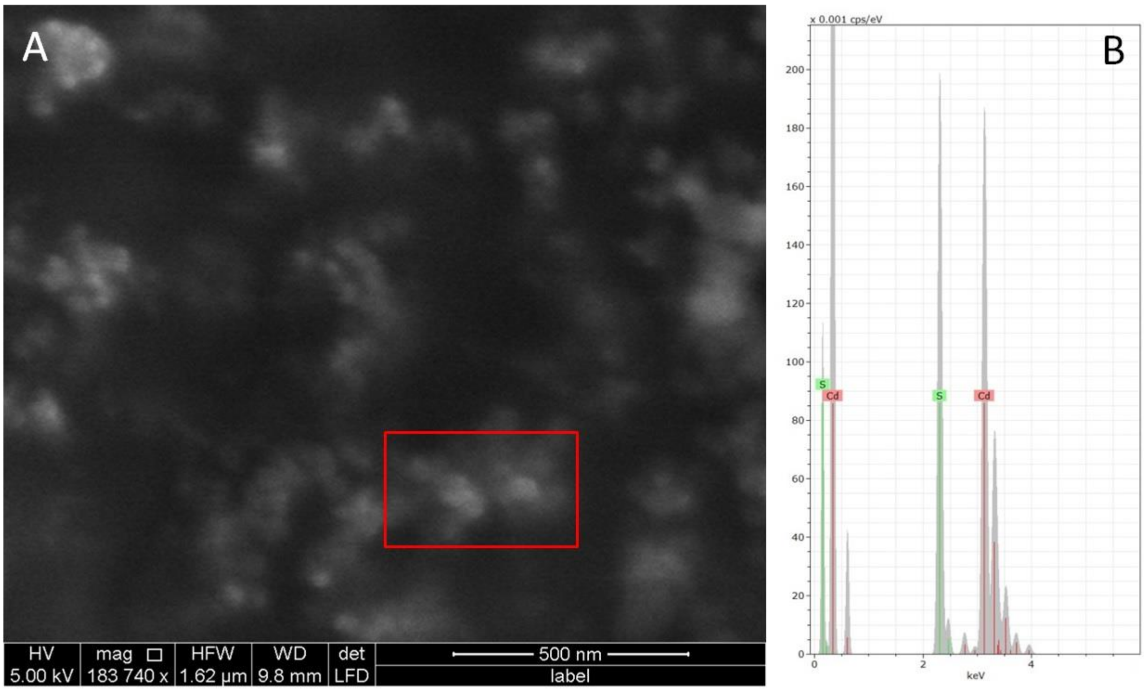

Figure 2. (A) ESEM image of the CdS QDs agglomerates. (B) X-ray spectra corresponding to the red rectangle in figure S2A.

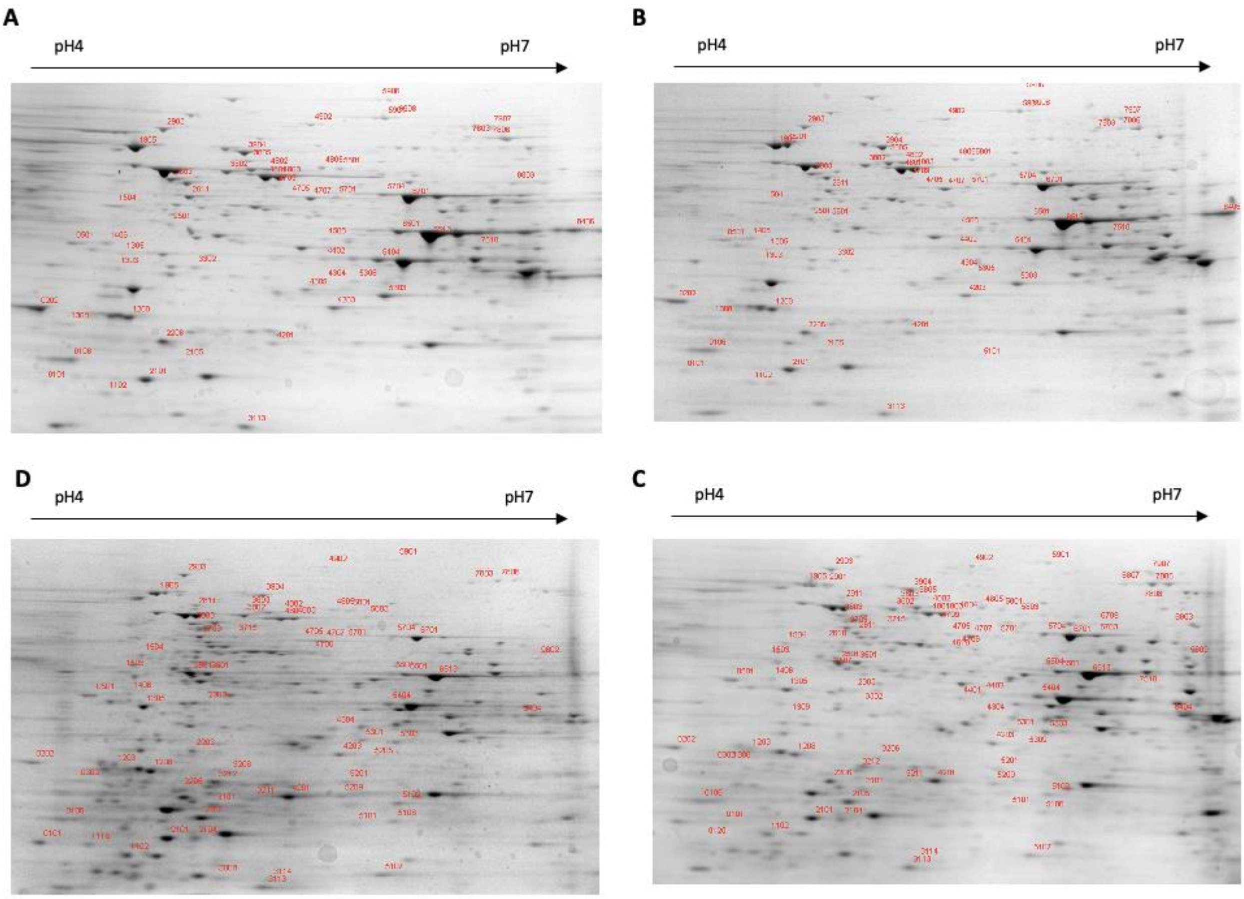

Figure 3. 2D-GE for the 9 h treatments. Yeast proteins (250 μg loaded) were separated in the first dimension over pH range 4–7 (11 cm strips) and on the second dimension by gel electrophoresis on 12% SDS-polyacrylamide gel. The gels were stained with Comassie brilliant blue. Red numbers in the gels indicate spots showing quantitative differences. These spots were cut from the gel and further identified by MS approach. Figures indicate: A) Control; B) nystatin; C) CdS QDs; D) Nystatin + CdS QDs.

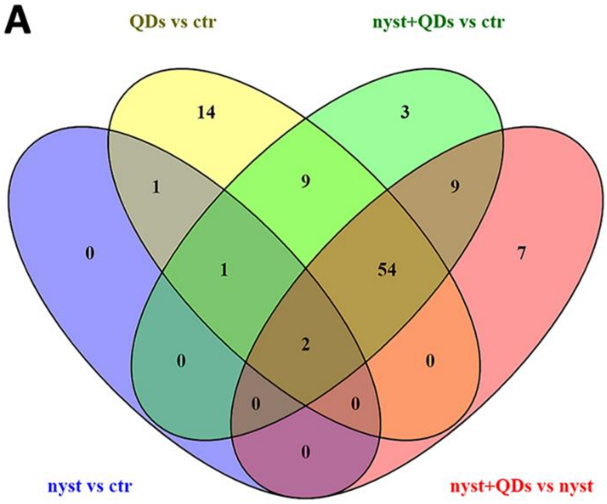

Figure 4. Venn diagrams for the differentially regulated proteins in all treatment conditions after 9 h treated with CdS QDs obtained with 2D-PAGE. The condition with only nystatin has almost no proteins in common with all the other conditions, whilst the conditions nyst + QDs vs nyst and QDs vs ctr have 54 proteins in common out of total 100. Fourteen proteins are typical of only the condition

QDs vs ctr. The conditions nyst + QDs vs ctr and nyst + QDs vs nyst have 63 proteins in common, while the condition nyst + QDs vs nyst alone has nine typical proteins. All the conditions together have in common two proteins.

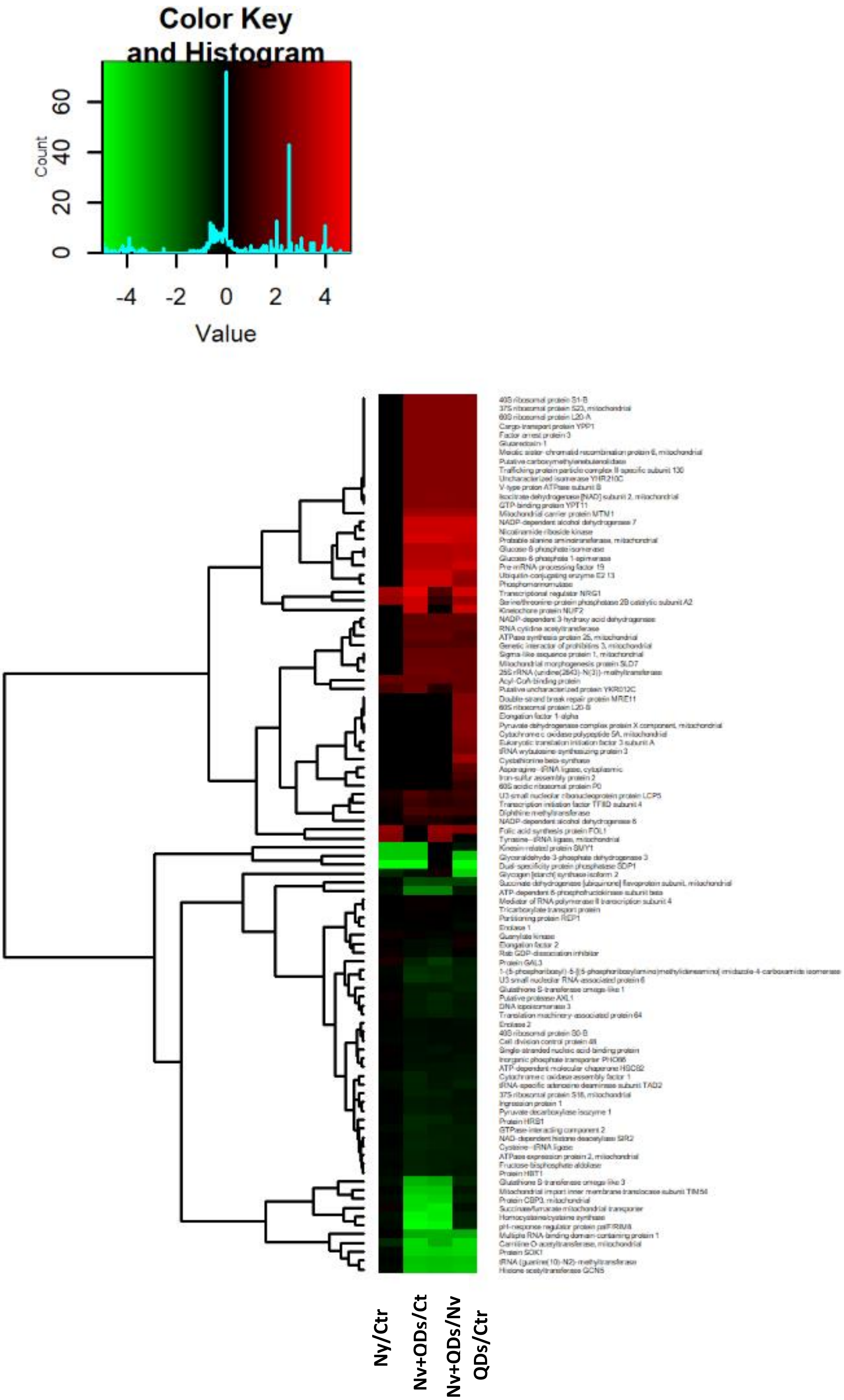

Color Key
and Histogram
Count
0
20
40
60
-4
-2
0
2
4
Value
40S ribosomal protein S1-B
37S ribosomal protein S23, mitochondrial
60S ribosomal protein L20-A
Cargo-transport protein YPP1
Factor arrest protein 3
Glutaredoxin-1
Meiotic sister-chromatid recombination protein 6, mitochondrial
Putative carboxymethylenebutenolidase
Trafficking protein particle complex II-specific subunit 130
Uncharacterized isomerase YHR210C
V-type proton ATPase subunit B
Isocitrate dehydrogenase [NAD] subunit 2, mitochondrial
GTP-binding protein YPT11
Mitochondrial carrier protein MTM1
NADP-dependent alcohol dehydrogenase 7
Nicotinamide riboside kinase
Probable alanine aminotransferase, mitochondrial
Glucose-6-phosphate isomerase
Glucose-6-phosphate 1-epimerase
Pre-mRNA-processing factor 19
Ubiquitin-conjugating enzyme E2 13
Phosphomannomutase
Transcriptional regulator NRG1
Serine/threonine-protein phosphatase 2B catalytic subunit A2
Kinetochore protein NUF2
NADP-dependent 3-hydroxy acid dehydrogenase
RNA cytidine acetyltransferase
ATPase synthesis protein 25, mitochondrial
Genetic interactor of prohibitins 3, mitochondrial
Sigma-like sequence protein 1, mitochondrial
Mitochondrial morphogenesis protein SLD7
25S rRNA (uridine(2843)-N(3))-methyltransferase
Acyl-CoA-binding protein
Putative uncharacterized protein YKR012C
Double-strand break repair protein MRE11
60S ribosomal protein L20-B
Elongation factor 1-alpha
Pyruvate dehydrogenase complex protein X component, mitochondrial
Cytochrome c oxidase polypeptide 5A, mitochondrial
Eukaryotic translation initiation factor 3 subunit A
tRNA wybutosine-synthesizing protein 3
Cystathionine beta-synthase
Asparagine--tRNA ligase, cytoplasmic
Iron-sulfur assembly protein 2
60S acidic ribosomal protein P0
U3 small nucleolar ribonucleoprotein protein LCP5
Transcription initiation factor TFIID subunit 4
Diphthine methyltransferase
NADP-dependent alcohol dehydrogenase 6
Folic acid synthesis protein FOL1
Tyrosine--tRNA ligase, mitochondrial
Kinesin-related protein SMY1
Glyceraldehyde-3-phosphate dehydrogenase 3
Dual-specificity protein phosphatase SDP1
Glycogen [starch] synthase isoform 2
Succinate dehydrogenase [ubiquinone] flavoprotein subunit, mitochondrial
ATP-dependent 6-phosphofructokinase subunit beta
Mediator of RNA polymerase II transcription subunit 4
Tricarboxylate transport protein
Partitioning protein REP1
Enolase 1
Guanylate kinase
Elongation factor 2
Rab GDP-dissociation inhibitor
Protein GAL3
1-(5-phosphoribosyl)-5-[(5-phosphoribosylamino)methylideneamino] imidazole-4-carboxamide isomerase
U3 small nucleolar RNA-associated protein 6
Glutathione S-transferase omega-like 1
Putative protease AXL1
DNA topoisomerase 3
Translation machinery-associated protein 64
Enolase 2
40S ribosomal protein S0-B
Cell division control protein 48
Single-stranded nucleic acid-binding protein
Inorganic phosphate transporter PHO86
ATP-dependent molecular chaperone HSC82
Cytochrome c oxidase assembly factor 1
tRNA-specific adenosine deaminase subunit TAD2
37S ribosomal protein S18, mitochondrial
Ingression protein 1
Pyruvate decarboxylase isozyme 1
Protein HRB1
GTPase-interacting component 2
NAD-dependent histone deacetylase SIR2
Cysteine--tRNA ligase
ATPase expression protein 2, mitochondrial
Fructose-bisphosphate aldolase
Protein HBT1
Glutathione S-transferase omega-like 3
Mitochondrial import inner membrane translocase subunit TIM54
Protein CBP3, mitochondrial
Succinate/fumarate mitochondrial transporter
Homocysteine/cysteine synthase
pH-response regulator protein palF/RIM8
Multiple RNA-binding domain-containing protein 1
Carnitine O-acetyltransferase, mitochondrial
Protein SOK1
tRNA (guanine(10)-N2)-methyltransferase
Histone acetyltransferase GCN5
Ny/Ctr
Nv+QDs/Ct
Nv+QDs/Nv
QDs/Ctr

Figure 5. Heat map of proteins altered at 9 h under treatment with CdSQDs found with 2D-PAGE. It is interesting to compare nystatin treatment with Crt with very few differences due to the treatment with nystatin. The same for the comparison of the treatment Nyst + QDs with Ctr and Nyst + QDs and the treatment with only nystatin (Nyst), both very similar. The condition QDs/Ctr was more complex than Nyst + QDs/Ctr.

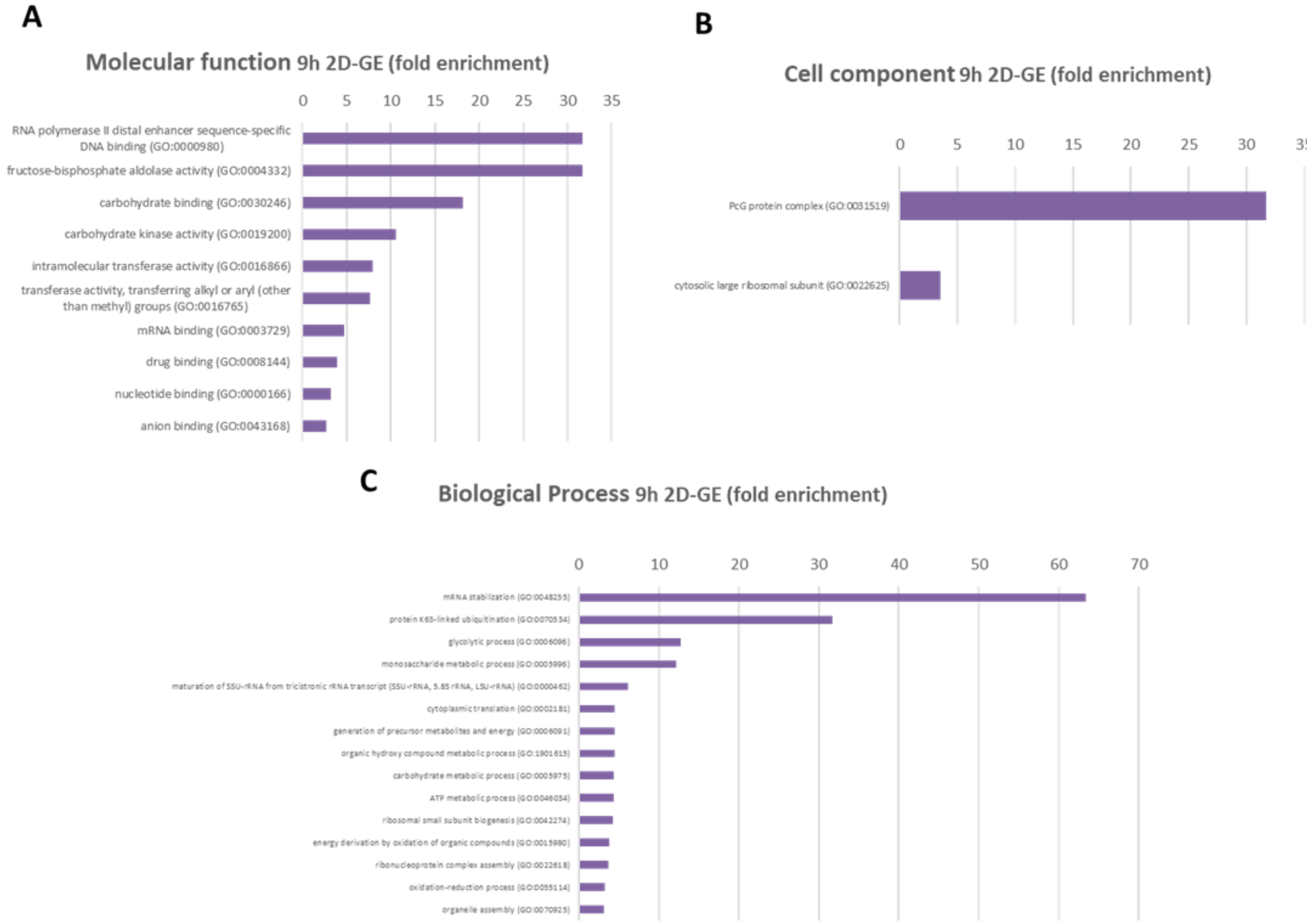

Figure 6. Gene ontology and enrichment analyses with fold enrichment = -log10 (Fisher's exact *p*-value) for A) molecular function; B) cell component; C) biological process obtained after 9 h treatment with CdS QDs utilising 2D-PAGE.

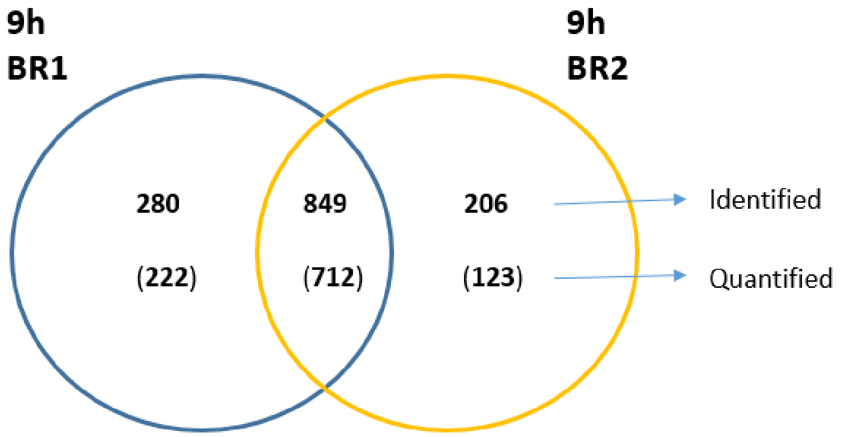

Figure 7. Venn diagram showing the number of unique proteins identified and quantified from the iTRAQ analysis of two biological replicates (BR1 and BR2) for the 9 h treatment corresponding to the four developmental stages.

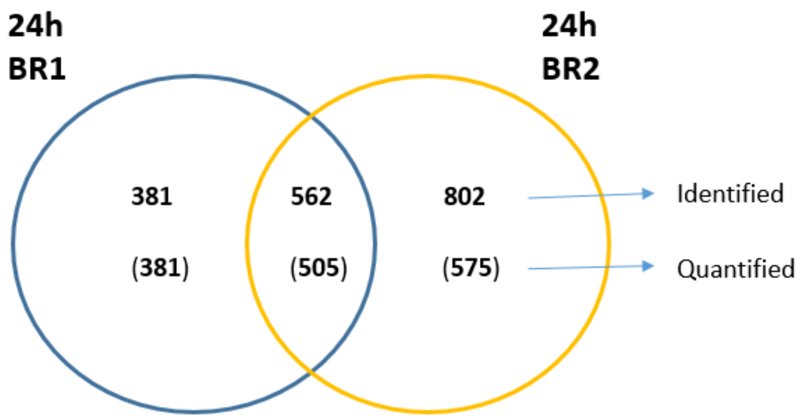

Figure 8. Venn diagram showing the number of unique proteins identified and quantified from the iTRAQ analysis of two biological replicates (BR1 and BR2) for the 24 h treatment corresponding to the four developmental stages.

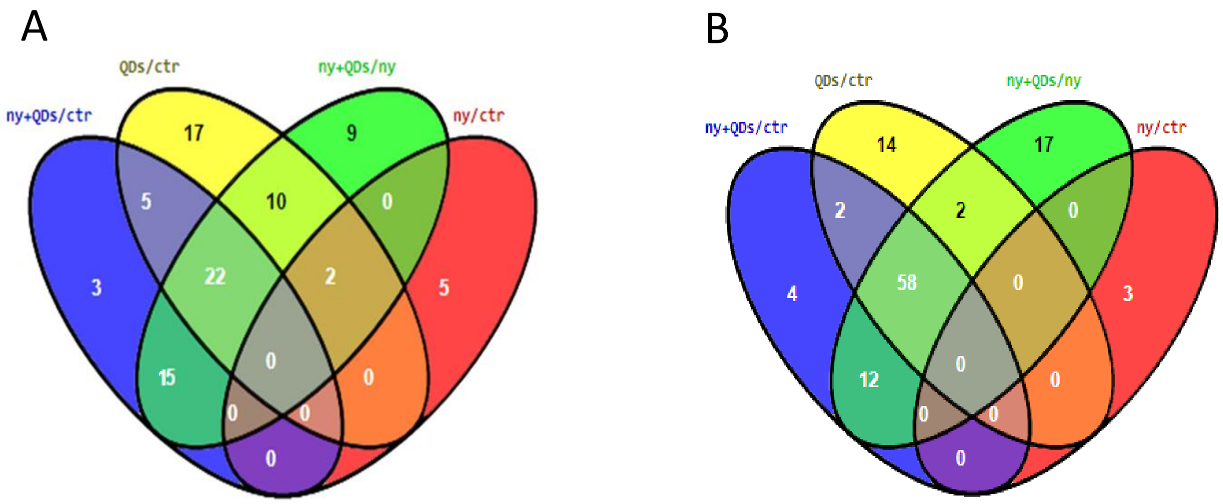

Figure 9. Venn diagrams for the differentially regulate proteins in all treatment conditions. A) 9 h iTRAQ; B) 24 h iTRAQ. The representations consider proteins consistently modulated in the two biological replicates BR1 and BR2.

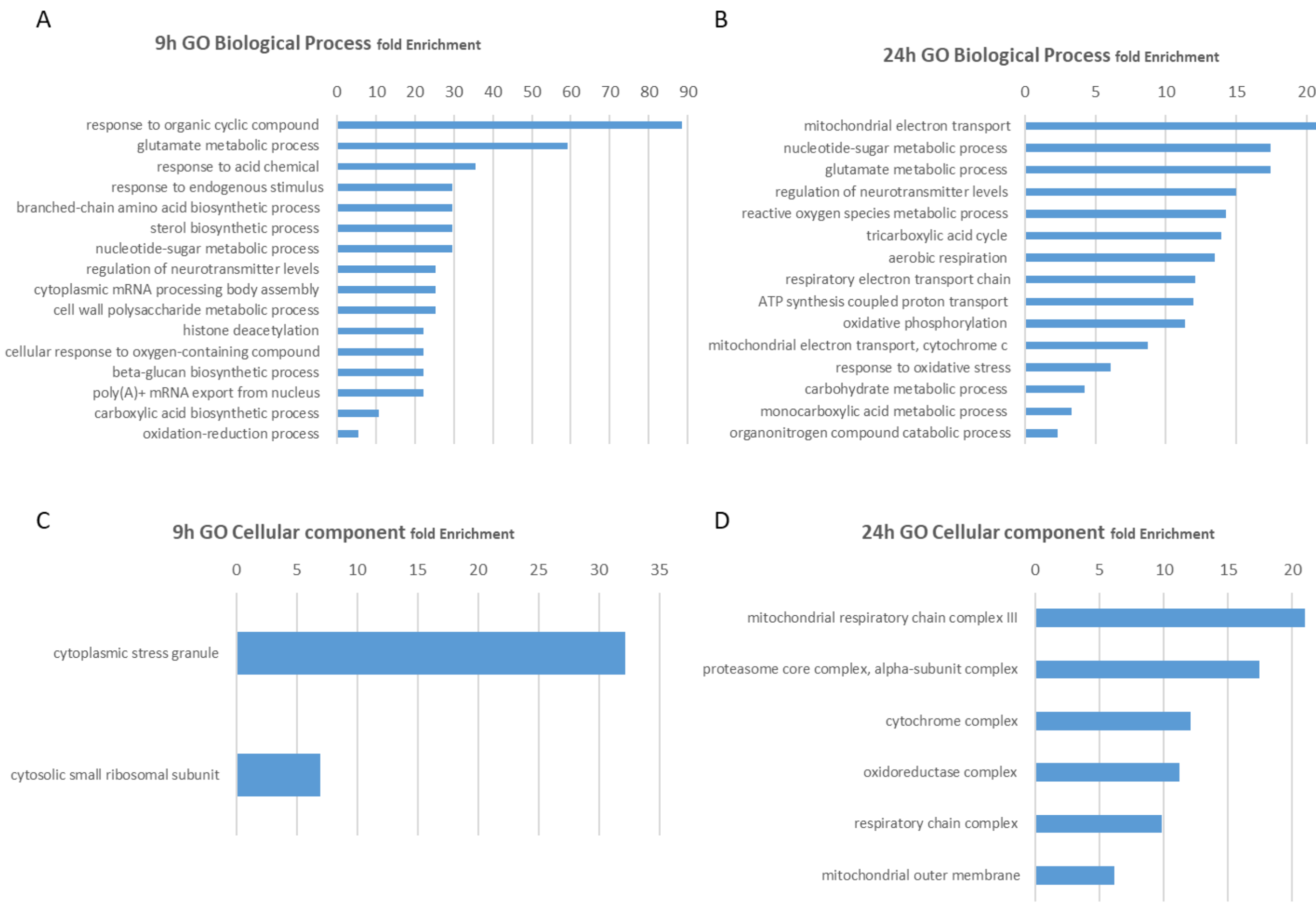

Figure 10. Gene ontology slim for iTRAQ, enrichment analyses with fold enrichment = -log10 (Fisher's exact *p*-value) for: A) 9 h biological process; B) 24 h treatments biological process; C) 9 h cell component; D) 24 h cell component.
